# Genetically engineered probiotic *E. coli* Nissle 1917 enhances protection against *Salmonella* via increased adhesion and systemic T-cell responses

**DOI:** 10.1038/s41522-026-01011-w

**Published:** 2026-05-21

**Authors:** Ewa Carolak, Joanna Czajkowska, Wiktoria Waszczuk, Agata Dutkiewicz, Anna Ewa Kedzierska, Krzysztof Grzymajlo

**Affiliations:** 1 Department of Biochemistry and Molecular Biology, Faculty of Veterinary Medicine, Wrocław University of Environmental and Life Sciences, Wrocław, Poland; 2 Department of Experimental Therapy, Hirszfeld Institute of Immunology and Experimental Therapy, Polish Academy of Sciences, Wrocław, Poland

**Keywords:** Immunology, Microbiology

## Abstract

The rise in antimicrobial resistance underscores the need for innovative strategies to combat gastrointestinal infections. Probiotics such as *Escherichia coli* Nissle 1917 (EcN) offer promising options, but the molecular mechanisms underlying their protective effects remain unclear. We introduce a G66R point mutation in FimH, creating a high-binding EcN variant that more effectively prevents *Salmonella* Typhimurium attachment and induces a distinct host transcriptional profile, shifting toward adaptive rather than innate inflammatory signaling. In vivo, EcN^G66R^ pretreatment significantly reduced intestinal colonization, fecal shedding, and systemic spread, and prevented splenic enlargement compared with EcN^WT^. Protection was associated with a marked expansion of CD4^+^ and CD8^+^ T cells, essential for clearing intracellular pathogens. EcN^G66R^ further enhanced “readiness” in the spleen under non-infected conditions, without adverse effects on host physiology. EcN^G66R^ thus functions as a dual-action probiotic—improving competitive exclusion while priming cytotoxic T-cell-mediated protection—and provides a promising platform for developing next-generation microbe-based therapies.

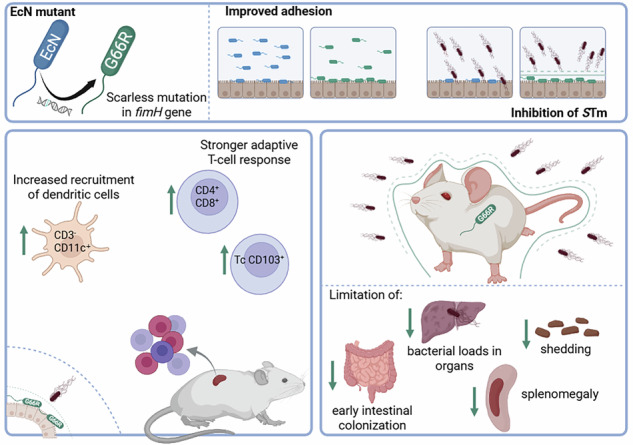

## Introduction

Gastrointestinal infections remain a major global health concern, with *Salmonella* spp. among the leading causes of foodborne illness worldwide. These pathogens cause a wide range of symptoms, from mild gastroenteritis to severe systemic disease, especially in vulnerable groups such as children, the elderly, and immunocompromised individuals. The infection starts with invasion of the intestines, then *Salmonella* spreads to distal tissues, including mesenteric lymph nodes, liver, and spleen^1^. The spleen, a vital lymphoid organ where immune responses to blood-borne pathogens are initiated, becomes a key site for bacterial replication during systemic salmonellosis^1^.

The host defense against *Salmonella* infection involves an interplay between immediate innate immunity and adaptive immune memory. Although initial bacterial control relies on rapid innate responses, long-term protection depends on establishing antigen-specific B and T cell populations capable of mounting recall responses upon re-exposure. The cellular immune response, mediated by helper CD4^+^ and cytotoxic CD8^+^ T cells, is particularly important for eliminating this facultative intracellular pathogen from infected macrophages and epithelial cells^2^.

With the increasing emergence of multidrug-resistant pathogens, conventional antibiotic therapy faces growing limitations, necessitating the development of innovative preventive and therapeutic strategies. Probiotic bacteria have gained significant attention as an effective approach to prevent or reduce gastrointestinal infections. Their beneficial effects occur through various mechanisms, including competitive exclusion for adhesion sites and nutrients, production of antimicrobial substances, and modulation of the host immune response^3^.

*Escherichia coli* Nissle 1917 (EcN) is one of the best-characterized probiotics, originally isolated from a German soldier who remained healthy during a severe shigellosis outbreak in 1917^4^. EcN (serotype O6:K5:H1) has been extensively studied and clinically applied for over a century^5–7^. EcN possesses unique genetic and phenotypic characteristics that distinguish it from pathogenic *E*. *coli* strains. It lacks typical virulence factors while expressing beneficial features such as microcin production, which inhibits enteropathogenic bacteria in the inflamed gut^8^. Additionally, EcN does not produce enterotoxins or cytotoxins^6^ and exhibits acid and bile tolerance, crucial for survival in the gastrointestinal tract^5^. Yet despite extensive clinical application and documented therapeutic benefits, the molecular mechanisms driving its protective effects against enteric pathogens remain only partially characterized^9^.

Bacterial adhesion to intestinal epithelial cells represents a critical determinant in the probiotic–pathogen competitive relationship. In *E. coli*, including probiotic strains, adhesion is primarily mediated by fimbrial structures^6^. Type 1 fimbriae (T1F) are among the most studied adhesive organelles, with the FimH adhesin located at their tip serving as the primary binding molecule. FimH is a mannose-specific lectin that recognizes and binds to mannosylated glycoproteins on the host cell surface. The binding properties of FimH can vary significantly between strains, with even single amino acid substitutions capable of dramatically altering binding affinity and specificity^10^. Therefore, enhancing FimH-mediated adhesion may strengthen a probiotic’s ability to compete with pathogens for epithelial binding sites, thus reinforcing colonization resistance. However, improved adhesion may also influence directly or indirectly other probiotic functions, including host signaling, immune interactions, and microbial fitness in the gut. FimH functions as an immunomodulatory molecule beyond its adhesive role. By acting as a direct TLR4 ligand, it activates MyD88-dependent NF-κB signaling and drives pro-inflammatory cytokine production, including IFN-β and IL-1β, in epithelial cells and macrophages^11–13^. FimH **s**timulates NK cell cytotoxicity, promotes dendritic cell maturation, and enhances antigen-specific T cell responses^14,15^. In the *Salmonella* infection model, FimH contributes directly to the induction of intestinal inflammation during bacterial invasion^16^.

The present study aimed to assess the effect of genetically enhanced EcN with improved adhesion properties. Among several point mutations that increase EcN binding to epithelial cell lines in vitro, we chose the G66R mutant in the FimH adhesin. We examined EcN’s high-binding phenotype in protecting against *Salmonella* Typhimurium infection and found that the mutant strain protects the host more effectively. We thoroughly characterized the phenotypic changes caused by the G66R mutation in the probiotic context and evaluated its influence on infection outcome, host immune responses, and protection against pathogen challenge.

## Results

### Point mutations in the fimH gene of *E. coli* Nissle 1917 improve its ability to attach to intestinal epithelial cell lines while causing minor alterations in host gene expression

**Fig. 1:**
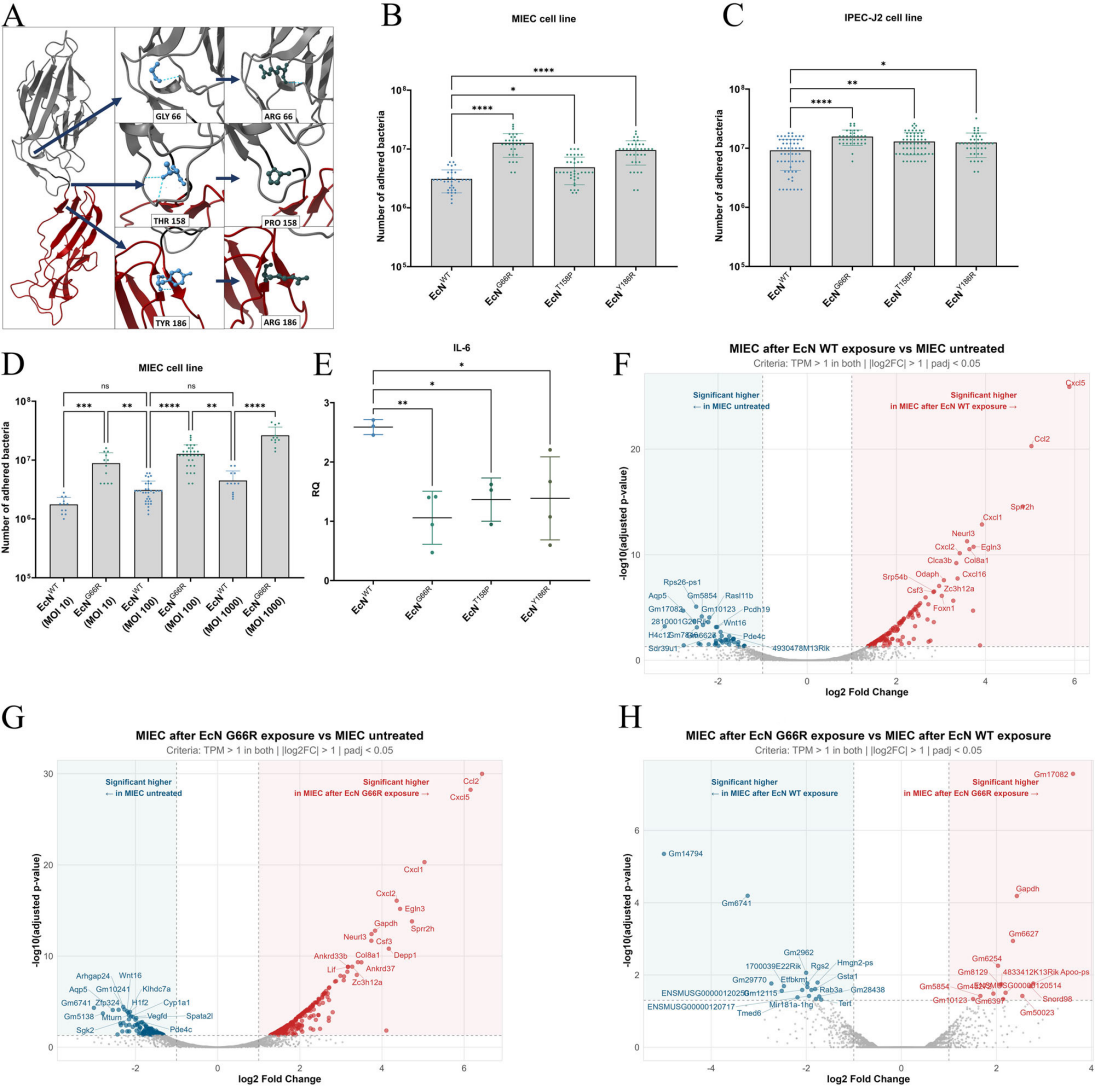
A point mutation in the *fimH* gene affects the binding properties of *E. coli* Nissle 1917 and modifies host cell response. **A** Structural visualization of introduced mutations in the *fimH* gene using ColabFold (implementation of AlphaFold2. Amino acid substitutions are highlighted on the protein structure (the pilin domain (gray), the lectin domain (red)). Original residues (blue), mutated residues (green), from top to bottom: G66R, T158P, Y186R. Binding of EcN and its mutants to mice (**B**) or porcine (**C**) intestinal epithelial cell lines at MOI 100. **D** Binding of EcN^WT^ and EcN^G66R^ to MIEC at MOI 10, 100, and 1000. **E** Relative expression of *IL-6* following incubation of the MIEC with *E. coli Nissle* and its fimH point mutants. Volcano plots showing (DEGs) between: **F** MIEC untreated versus MIEC after exposure to EcN^WT^; **G** MIEC untreated versus MIEC after exposure to EcN^G66R^; **H** MIEC after exposure to EcN^G66R^ and the MIEC after exposure to EcN^WT^. Genes significantly up-regulated—red (right side), genes significantly down-regulated—blue (left side). Not significant genes—gray. Labeled genes—top 20 most significant by *p*-value. Vertical dashed lines mark the ±1log₂FC threshold, and the horizontal dashed line indicates the −log₁₀(padj) threshold. Statistical differences between strains and various conditions were analyzed by one-way ANOVA with Tukey’s post-hoc test, preceded by a normality test, using GraphPad Prism version 11.0.0 for Windows, GraphPad Software, Boston, MA, USA. www.graphpad.com.

One potential mechanism of probiotic action is competing with pathogens for environmental niches and binding sites on host intestinal cells. Here, we selected high-binding phenotypes of pathogenic *E. coli*^17–19^, and introduced point mutations responsible for those phenotypes into the *fimH* gene (G66R, T158P, and Y186R) of *E. coli* Nissle 1917 (EcN) (Table 1, Fig. 1A). Mutant strains showed no detectable differences in growth dynamics compared to the wild-type strain (data not shown). Subsequently, we assessed its adhesion properties using two intestinal epithelial cell lines (Fig. 1B, C). The number of adhered bacteria increased significantly, approximately 4.1 times for EcN^G66R^ (*p* < 0.0001), 1.6 times for T158P (*p* < 0.05), and 3.1 times for Y186R (*p* < 0.001) compared to the EcN^WT^ strain during interaction with the MIEC cell line (Fig. 1B). Notably, this binding pattern stayed consistent during interaction with the IPEC-J2 cell line (Fig. 1C). To determine whether the improved binding could be replicated by simply increasing the bacterial dose, we conducted adhesion assays using different MOI levels for the mutant with the most significant difference in adhesion. Importantly, even at elevated MOI, EcN^WT^ did not achieve binding levels comparable to the EcN^G66R^ (Fig. 1D). The probiotic mechanisms of action involve the activation or silencing of innate responses in intestinal cells^17,18,20^. Therefore, we assessed whether the altered binding pattern influences the expression levels of selected cytokines associated with gastrointestinal infections. Interestingly, among the tested cytokines, the only statistically significant difference was observed for the *IL-6* gene, with decreased expression in the MIEC cell line in the presence of the selected mutant compared to EcN^WT^ (Fig.1E) (*p* < 0.05). Expression levels of IL-1β, IL-10, IL-12, IL-19, and IFN-γ showed no significant differences between mutant and WT treatments (Supplementary Fig. 3A). These minimal differences led us to investigate how the EcN^G66R^ affects the overall MIEC transcriptional response using RNA sequencing (RNA-seq). After DEG analysis, we observed 143 and 43 genes significantly up- and downregulated, respectively, in EcN^WT^ compared to the untreated control (Fig. 1F). The EcN^G66R^ mutant increased the expression of 280 genes and decreased the expression of 119 genes compared to untreated cells (Fig. 1G). Venn diagram analysis showed overlap in responses between the two strains: 129 genes were upregulated by both, with 151 genes uniquely upregulated by EcN^G66R^ and only 14 by EcN^WT^. Only 16 genes were commonly suppressed, while 103 genes were uniquely downregulated by EcN^G66R^ and 27 genes by EcN^WT^ (Supplementary Fig. 3B, Supplementary Table 4).

**Table 1:** Bacterial strains used in this study

Name	Strain	Mutation in *fimH*	Source
EcN^WT^	*Escherichia coli* Nissle 1917	Wild-type	Schierack lab strain collection
EcN^G66R^	*Escherichia coli* Nissle 1917 with a point mutation (G66R) in *fimH* gene	66 (Gly-Arg) GGC to CGC; originally reported in ref. ^17^	This study
EcN^TI58P^	*Escherichia coli* Nissle 1917 with a point mutation (T158P) in *fimH* gene	158 (Thr-Pro) ACT to CCT; originally reported in ref. ^19^	This study
EcN^Y186R^	*Escherichia coli* Nissle 1917 with a point mutation (Y186R) in *fimH* gene	186 (Tyr-Arg) TAT to CGT, originally reported in ref. ^18^	This study
*S*. Tm^WT^	*S*. Typhimurium SL1344	Wild-type	Monack lab strain collection
S. Tm^WT_mCherry^	*S*. Typhimurium SL1344 with mCherry plasmid	n/a	This study
*E. coli* ^DH5α λpir^	*Escherichia coli* DH5α with integrated conjugal transfer functions	n/a	Monack lab strain collection
*E. coli* ^S17-1 λpir^	*E. coli S17-1 with integrated conjugal transfer functions*	n/a	Monack lab strain collection
EcN^WT^*-*EryR	*Escherichia coli Nissle* 1917; EryR	Wild-type	This study
EcN^G66R^*-*EryR	*Escherichia coli* Nissle 1917 with a point mutation (G66R) in *fimH* gene; EryR	66 (Gly-Arg)	This study
EcN^WT^*-NalR*	*Escherichia coli* Nissle 1917; NalR	Wild-type	This study
EcN^G66R^*-NalR*	*Escherichia coli* Nissle 1917 with a point mutation (G66R) in *fimH* gene; NalR	66 (Gly-Arg)	This study

To better illustrate that the differences between the probiotic strains are modest, significant DEGs were ranked by the standard deviation of log2 (fold change). The top 50 most variable genes for each condition were plotted (Supplementary Fig. 3C), revealing that, even among these most strongly affected genes, expression changes rarely exceeded ~2-fold differences between strains. To compare the transcriptional programs triggered by EcN^WT^ and EcN^G66R^, we conducted direct differential expression analysis. We identified 14 genes with significantly higher expression in EcN^G66R^-treated cells, primarily pseudogenes and ncRNAs, and 17 genes with higher expression in EcN^WT^-treated cells (Fig. 1H/Supplementary Table 6). EcN^G66R^ mainly upregulated GAPDH and non-coding RNAs. EcN^WT^ induced a coordinated cellular defense program characterized by antioxidant protection, regulated inflammatory signaling, and improved protein trafficking. Importantly, when we specifically examined immune-related genes, we found no significant differences between the two strains: among 765 immune-associated genes analyzed, indicating that the FimH G66R mutation does not fundamentally alter the core immunological transcriptional response in MIEC cells.

### EcN^G66R^ and EcN^T158P^ mutants inhibit *Salmonella* Typhimurium cell attachment more effectively than EcN^WT^

**Fig. 2:**
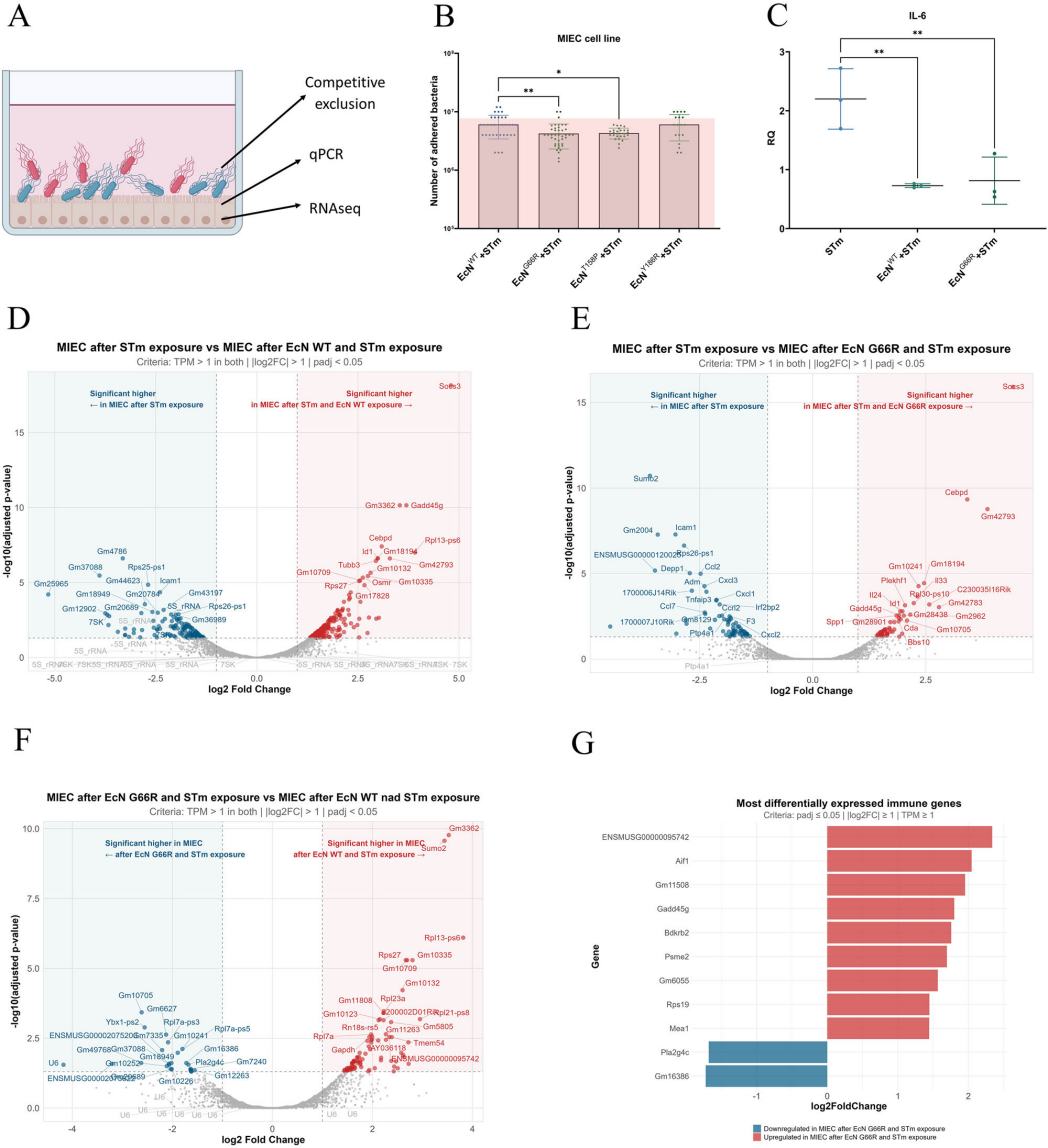
Probiotics with enhanced adhesion properties affect *Salmonella*–host cell interactions. **A** Schematic illustrating the experimental workflow for the competitive exclusion assay and subsequent analysis. Created in BioRender. Grzymajlo, K. (2026) https://BioRender.com/zhk97cx. **B** Inhibition of *S*Tm adhesion to the MIEC preincubated with different EcN variants. The bars show the reduction in *S*Tm adhesion level, and the background shading indicates the adhesion level of *S*Tm alone. **C** Relative expression of IL-6 after incubation of the MIEC with EcN^WT^ and EcN^G66R^ followed by infection with *S*Tm. Volcano plot showing DEGs between: MIEC after exposure to *S*Tm versus: **D** MIEC after exposure to EcN^WT^ and then *S*Tm; **E** MIEC after exposure to EcN^G66R^ and then *S*Tm; **F** MIEC after exposure to EcN^WT^ and *S*Tm versus the MIEC after exposure to EcNG^66R^ and *S*Tm. Genes significantly up-regulated—red (right side), genes significantly down-regulated—blue (left side). Not significant genes—gray. Labeled genes- top 20 most significant by *p*-value. Vertical dashed lines indicate the ±1log₂FC threshold, and the horizontal dashed line indicates the −log₁₀(padj) threshold. **G** Top differentially expressed immunological genes identified by RNA-seq analysis in the MIEC after exposure to EcN^WT^ and *S*Tm versus the MIEC after exposure to EcN^G66R^ and *S*Tm. The bar plot displays immune-related genes - upregulated (red) and downregulated (blue), with the highest absolute log₂ fold change. Statistical significance was assessed using a one-way ANOVA with Tukey’s post hoc test for data that met the assumptions of normality, whereas the Kruskal–Wallis test with Dunn’s post hoc test was used when the data did not meet these assumptions. Statistical analyses were performed using GraphPad Prism version 11.0.0 for Windows, GraphPad Software, Boston, MA, USA. www.graphpad.com.

Given the critical role of competitive exclusion in probiotic function, we re-evaluated all three mutants to test whether their increased adhesion improves protection against pathogens (Fig. 2A). Pretreatment with EcN^G66R^ and EcN^T158P^ significantly reduced *S*Tm binding compared with EcN^WT^ (Fig. 2B), with EcN^G66R^ showing the most potent protective effect. This consistent superior performance of the EcN^G66R^ mutant across both adhesion and pathogen-exclusion assays reinforced our decision to select this variant for further investigation. To assess potential immune modulation, the expression of cytokines previously examined in probiotic-treated cells was re-evaluated by qPCR in MIEC cells infected with *S*Tm, with or without probiotic pretreatment (Supplementary Fig. 4A). Notably, pre-treatment with either EcN^WT^ or EcN^G66R^ mutants significantly reduced *S*Tm-induced IL-6 expression compared to *S*Tm infection alone (Fig. 2C) (*p* < 0.01). However, no significant differences in IL-6 expression were observed between EcN^WT^ and mutant EcN^G66R^ pre-treatments in the presence of *S*Tm (Fig. 2C). Expression levels of IL-1β, IL-10, IL-12, IL-19, and IFN-γ remained unchanged (Supplementary Fig. 4A). To identify genes uniquely affected by each treatment condition, we compared all three setups (*S*Tm alone, EcN^WT^ + *S*Tm, EcN^G66R^ + *S*Tm) against uninfected control cells (Fig. 2D, E). Venn diagram analysis revealed sets of uniquely regulated genes: 234 genes were downregulated in EcN^WT^ + *S*Tm, 114 in EcN^G66R^ + *S*Tm, and 18 in *S*Tm alone. Similarly, 170 genes were upregulated in EcN^WT^ + *S*Tm, 66 in EcN^G66R^ + *S*Tm, and 32 in *S*Tm alone (Supplementary Fig. 4B). This suggests that pre-treatment with either probiotic strain significantly alters the cellular response to *Salmonella* infection. Further analysis of gene expression variability across the three infection conditions highlighted the transcripts that responded most dynamically to treatment. The top 50 differentially expressed genes were selected based on the magnitude of fold-change variation (Supplementary Fig. 4C). Notably, several inflammatory and stress-response genes, including *Ccl2*, *Cxcl1*, *Egln3*, *Cxcl2*, and *Cebpd*, showed the strongest modulation. When directly compared, infection with *S*Tm in the presence of EcN^WT^ resulted in 24 upregulated genes, whereas *S*Tm infection in the presence of EcN^G66R^ resulted in 84 upregulated genes (Fig. 2F). Analysis of immune-related genes specifically revealed that among 791 immunological genes examined, 11 showed significant differential expression between the two treatment groups, with nine genes upregulated and two genes downregulated in EcN^G66R^ + *S*Tm compared to EcN^WT^ + *S*Tm (Fig. 2G).

### EcN^G66R^ offers improved protection against systemic salmonellosis by reducing bacterial colonization and preventing infection-associated pathology

**Fig. 3:**
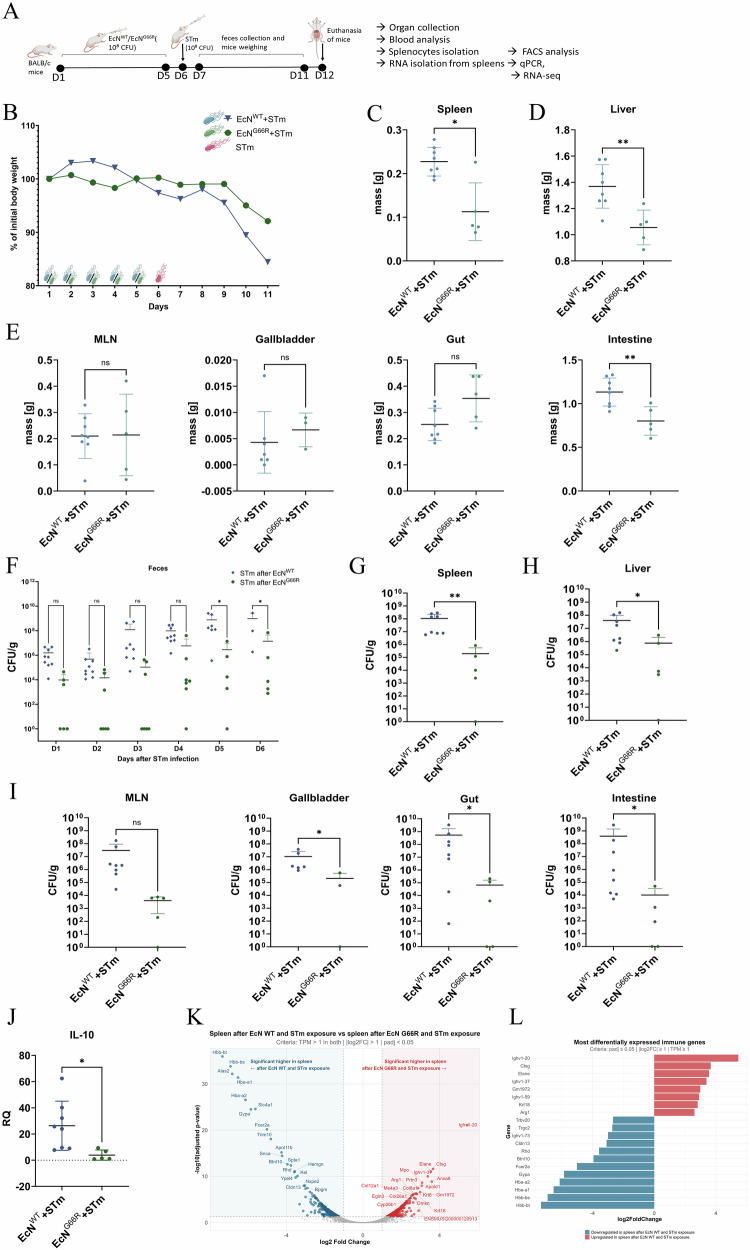
EcN^G66R^ protects BALB/c mice more efficiently against *Salmonella* Typhimurium infection. **A** Schematic representation of the animal infection experiment workflow. Created in BioRender. Grzymajlo, K. (2026) https://BioRender.com/zhk97cx. **B** Percentage change in body weight following *S*Tm infection in BALB/c mice pretreated with either EcN^WT^ (blue) or EcN^G66R^ (green). (Top left) Comparison of organ weights (in grams) from mice infected with *S*Tm mice pretreated with EcN^WT^ (blue)(*n* = 8) or EcN^G66R^ (green)(*n* = 5). **C** Spleen, **D** liver, **E** mesenteric lymph nodes (MLN), gallbladder, gut, and intestine. Each data point represents an individual mouse, and horizontal lines indicate the mean ± SEM. **F** *S*Tm bacterial counts in feces (CFU/g) over 6 days post-infection (D1–D6) comparing EcN^WT^(blue dots) and EcN^G66R^ (green dots) treatments. Bacterial loads in organs: **G** spleen, **H** liver, **I** MLM, gallbladder, gut, and intestine. Data are expressed as CFU/g, with each symbol representing an individual animal—EcN^WT^ (blue dots)(*n *= 8) and EcN^G66R^ (green dots)(*n *= 5), and bars indicating mean ± SEM. For logarithmic presentation, uncolonized samples (no detectable bacteria) were assigned a threshold value of 1 CFU/g. **J** Relative expression of IL-10 in the spleen of mice post-infection with *S*Tm with pretreatment with EcN^WT^ (blue) and EcN^G66R^ (green). **K** Volcano plot showing DEGs between spleens from mice pretreated with EcN^WT^ + *S*Tm versus EcN^G66R^ + *S*Tm. Genes with TPM > 1 in both groups, |log₂(fold change)| > 1, and adjusted *p*-value < 0.05 are plotted. Genes significantly up-regulated (red) (right side) and genes significantly down-regulated (blue) (left side). Not significant genes—gray. Labeled genes—top 20 most significant by *p*-value. Vertical dashed lines indicate the ±1 log₂FC threshold, and the horizontal dashed line indicates the -log₁₀(padj) threshold. **L** Top differentially expressed immunological genes identified by RNA-seq analysis in spleens from mice pretreated with EcN^WT^ versus EcN^G66R^, both followed by *S*Tm infection. The bar plot shows the top 20 immune-related genes with the highest absolute log₂ fold change between the experimental conditions analyzed. Only genes with adjusted *p* ≤ 0.05, |log₂FC| ≥ 1, and TPM ≥ 1 were included. Red bars indicate genes upregulated under the tested condition, while blue bars represent genes downregulated in comparison. Statistical comparisons were performed using: 2-way ANOVA with multiple comparison test (*F*), unpaired Student’s *t*-test for data that met the assumptions of normality, and Mann–Whitney for the data that did not meet the assumptions of normality (**C**–**E**, **G**–**I**), after the removal of outliers (ROUT, *Q* = 1%), using GraphPad Prism version 11.0.0 for Windows, GraphPad Software, Boston, MA, USA, www.graphpad.com. The number of mice per group varies from 6 to 9.

Based on the in vitro effects shown by EcN^G66R^, we tested whether the mutant provides better protection for the host than EcN^WT^ in a *Salmonella-*susceptible BALB/c mouse model. We administered a high dose (10^8^) of the pathogen by oral gavage to induce systemic infection via the gastrointestinal route (Fig. 3A). Six days after infection, mice in both groups showed symptoms of salmonellosis (e.g., reduced appetite, weight loss, ruffled fur, hunched posture, lethargy), but mice pre-treated with EcN^G66R^ showed milder clinical signs throughout the infection: they had better coat condition, more natural posture, and higher spontaneous activity compared to EcN^WT^-pretreated animals. Blood analysis revealed significantly higher glucose levels in the mice pre-treated with EcN^G66R^ and then infected with *S*Tm (average 6.5 mmol/l) compared to the EcN^WT^ + *S*Tm group (average 3.1 mmol/l; *p* < 0.05) (Supplementary Fig. 5A). The EcN^G66R^ + *S*Tm group also had significantly higher magnesium levels (average 1.4 versus 1.0 mmol/l in EcN^WT^ + *S*Tm; *p* < 0.05), while potassium, sodium, and iron levels showed trends toward differences between groups, but these did not reach statistical significance (Supplementary Fig. 5A).

In the EcN^G66R^-treated group, the average mouse weight six days after *S*Tm infection dropped to ~95% of the initial weight, whereas in the EcN^WT^-treated group, it was about 84% (Fig. 3B). Importantly, mice pre-treated with the EcN^G66R^ mutant did not develop splenomegaly, a typical syndrome of systemic salmonellosis^21^, in contrast to the EcN^WT^ pre-treated group (Fig. 3C). In this group, we also observed an enlarged liver and intestine mass compared to the EcN^G66R^ group (Fig. 3D, E). No differences were detected in the mass of the gallbladder, lymph nodes, or gut (Fig. 3E).

During the first 3 days after infection, most mice in the EcN^G66R^ group did not exhibit fecal shedding, whereas all mice in the EcN^WT^-treated group shed feces from the first day. Over the following days (D5, D6 pi), shedding levels were significantly lower (*p* < 0.05) in the EcN^G66R^ group compared to EcN^WT^ (Fig. 3F). Further validation of the protective effect of EcN^G66R^ was performed by 16S rRNA gene sequencing of fecal samples and intestines from infected mice (Supplementary Table 5A, B). At day 1 post-infection, *S*Tm was detected only in EcN^WT^-treated mice (6 reads, 0.01039%), while EcN^G66R^-treated mice showed none. By day 4 post-infection, the difference widened: *Salmonella* abundance in EcN^WT^ + *S*Tm feces reached 5.823% (335 reads), whereas in EcN^G66R^ + *S*Tm mice, *Salmonella* constituted only 0.1503% (88 reads)—an ~39-fold. Total fecal *Enterobacteriaceae* followed the same pattern, reaching 0.913% (536 reads) in EcN^WT^ + *S*Tm (driven primarily by *Salmonella* expansion) versus 0.2107% (125 reads) in EcN^G66R^ + *S*Tm (~4.3-fold reduction) at day 4. Analysis of intestinal tissue colonization at day 6 post-infection confirmed the profound protective effect of EcN^G66R^ (Supplementary Table 5B). EcN^WT^ + *S*Tm mice showed substantial *Salmonella* colonization (3997 reads; 8.391%), indicating robust mucosal colonization, whereas EcN^G66R^ + *S*Tm mice had only three reads (0.00554%)—a ~1500-fold reduction. Total *Enterobacteriaceae* abundance mirrored this pattern: 17.55% in EcN^WT^ + *S*Tm versus only 0.02618% in EcN^G66R^ + *S*Tm, representing an ~670-fold decrease. Differences in overall microbial composition between groups were more noticeable in intestinal tissue than in fecal samples, where family-level profiles remained broadly similar between EcN^WT^ + STm and EcN^G66R^ + STm animals across all time points (Supplementary Fig. 7A, B). In intestinal tissue, both groups were dominated by *Muribaculaceae*, *Lachnospiraceae*, and *Lactobacillaceae*, and the main difference between groups was the relative abundance of *Enterobacteriaceae*, which was significantly higher in the EcN^WT^ + STm group, consistent with the pathogen colonization data above. In our experimental model, pretreatment with the EcN^G66R^ mutant resulted in a significant decrease in *Salmonella* burden across most tested tissues compared with EcN^WT^ pretreatment (Fig. 3G–I). In the spleen, bacterial levels were reduced by ~7300-fold (Fig. 3G)(*p* < 0.01), while in the liver fell 1700-fold (Fig. 3H)(*p* < 0.05). Reductions occurred in the MLN (350-fold; ns), gallbladder (60-fold; *p* < 0.05), and intestine (5800-fold; *p* < 0.05) (Fig. 3I). In the gut, bacterial loads decreased 13,000-fold (*p* < 0.05). Importantly, several mice in the EcN^G66R^-pretreated group showed a complete absence of *Salmonella* in multiple organs. In contrast, all mice in the EcN^WT^-pretreated group had detectable bacterial colonization in all examined tissues. qPCR analysis of infected spleens showed no significant differences in IL-6, IL-15, and IFN-γ expression between EcN^WT^ + *S*Tm and EcN^G66R^ + *S*Tm groups, while IL-10 was significantly higher in EcN^WT^ + *S*Tm mice (*p* < 0.05) (Fig. 3J, Supplementary Fig. 5B).

To understand the molecular basis for superior protection conferred by EcN^G66R^, including reduced organ colonization and prevention of splenomegaly, we performed RNA-seq analysis of spleens from BALB/c mice pretreated with either EcN^WT^ or EcN^G66R^ mutant, followed by *S*Tm infection. The transcriptomic analysis revealed differences between the two groups, with 695 differentially expressed genes (369 upregulated and 326 downregulated in EcN^WT^ + *S*Tm relative to EcN^G66R^ + *S*Tm) out of 27,466 total genes analyzed (Fig. 3K). Analysis of immune-related genes identified 1504 candidates, of which 223 were differentially expressed between treatment groups (Fig. 3L). The EcN^G66R^ + *S*Tm group upregulated 125 immune genes, while EcN^WT^ + *S*Tm upregulated 98, among which we identified a core neutrophil effector program—*Ctsg* (cathepsin G), *Elane* (neutrophil elastase), *Mpo* (myeloperoxidase), and *Prtn3* (proteinase 3)—key components of neutrophil extracellular traps (NETs) and bacterial killing. In addition, EcN^WT^ + *S*Tm mice displayed strong induction of the neutrophil-recruiting chemokines *Cxcl2* and *Cxcl3*, potent chemoattractants and central mediators of acute inflammation. Additional inflammatory chemokines and cytokines, including *Cxcl1*, *Cxcl11*, *Cxcl16*, *Ifng*, *IL-1b*, and *IL-10*, showed increased expression trends (*p* < 0.05 but padj > 0.05). Upregulation was further observed for *Selp* (P-selectin), *Itga2b* (integrin α2b), *Arg1* (arginase 1—typically associated with M2 macrophage polarization and immunoregulation), and *Ms4a3*.

In contrast, the 125 immune genes upregulated in EcN^G66R^ + *S*Tm mice revealed a different immunological strategy. EcN^G66R^ + *S*Tm mice showed significant upregulation of CD8^+^ T-cell markers (*Cd8a*, *Cd8b1*) and the T-cell/NK-cell recruiting chemokine *Xcl1* (lymphotactin) along with its receptor *Xcr1*. The response in EcN^G66R^ + *S*Tm mice was dominated by extensive T-cell activation, characterized by increased expression of multiple T-cell receptor chains (*Trbv1*, *Trbv13-1*, *Trbv13-2*, *Trbv14*, *Trbv19*, *Trbv20*, *Trgc2*, *Trgv1*, *Trbc1*, *Trbc2*, *Trac*, *Trav14d-3-dv8*), co-receptors (*Cd3d, Cd3e, Cd3g, Cd5, Cd7, Cd28*), and transcriptional regulators (*Bcl11b*, *Eomes*, *Lef1*, *Bach2*, *Themis*). The upregulation trend of IL-15 (padj = 0.084, *p* < 0.05), a critical cytokine for CD8^+^ T-cell and NK cell survival and proliferation. B-cell responses were likewise enhanced, with elevated expression of *Cd19*, *Cd22*, *Cd79a*, *Cd79b*, *Ms4a1* (CD20), *Fcer2a* (CD23), *Cr2* (CD21), and several immunoglobulin genes (*Ighv1-34*, *Ighv1-47*, *Ighv1-63*, *Ighv1-73*, *Ighv5-15*, *Ighv9-3*), with *Igkv2-112* maintaining its upregulation from the condition lacking *S*Tm. Increased expression of *Spib*, *Ikzf3*, and *Bank1* further supports B-cell activation. Additionally, EcN^G66R^ + *S*Tm mice showed a strong induction of interferon-stimulated genes (*Mx1*, *Mx2*, *Ifi206*, *Ifi208*, *Ifit3*, *Ifit3b*, *Ifit1bl1*, *Oas1b*), as well as chemokines *Ccl21a* (secondary lymphoid chemokine) and *Ccl24* (eosinophil chemotactic protein).

### EcN^WT^ and EcN^G66R^ pretreatment elicits immune modulation and differential protection against *Salmonella* Typhimurium infection

**Fig. 4:**
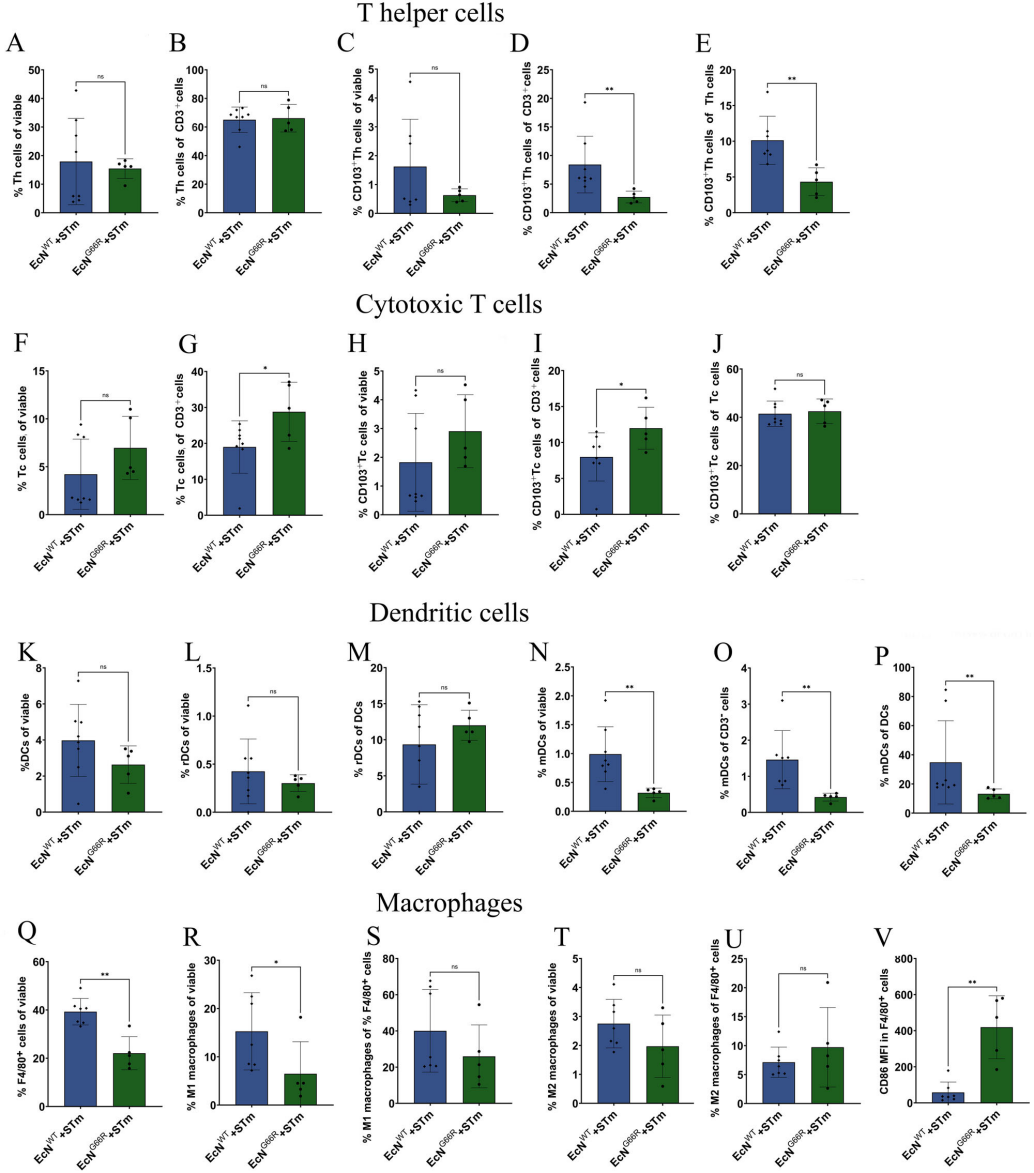
EcN^G66R^ modifies the immune response in mice infected with *Salmonella.* The data shown the percentages of : T helper (Th, CD3^+^CD4^+^) cells **A** among viable cells, **B** among CD3^+^ cells; CD103^+^ Th cells (CD3^+^CD4^+^CD103^+^), **C** among viable cells, **D** among CD3^+^ cells and **E** among Th cells; T cytotoxic (Tc, CD3^+^CD8a^+^) cells **F** among viable cells and **G** among CD3^+^ cells; CD103^+^ Tc cells (CD3^+^CD8a^+^CD103^+^), **H** among viable cells, **I** among CD3^+^ cells, **J** among Tc cells, **K** dendritic cells (DCs, CD3^−^CD11c^+^) among viable cells; resident dendritic cells (rDCs, CD3^−^CD11c^+^CD8a^+^), **L** among viable cells and **M** among DCs; migratory dendritic cells (mDCs, CD3^−^CD11c^+^CD103^+^), **N** among viable cells, **O** among CD3^−^ and **P** among DCs; **Q** F4/80^+^ macrophages (CD3^−^F4/80^+^) among viable cells; M1 macrophages (CD3^−^F4/80^+^CD86^+^), **R** among viable and **S** F4/80^+^ macrophages; M2 macrophages (CD3^−^F4/80^+^CD206^+^), **T** among viable cells and among, **U** F4/80^+^ macrophages, and **V** the specific fluorescence intensity of CD86 on the surface on F4/80^+^ macrophages in the spleen cells after *Salmonella* infection in mice pretreated with EcN^WT^ (*n* = 7) and EcN^G66R^ (*n* = 7). Values are presented as mean ± SEM. Statistical significance: for normally distributed data analyzed with an unpaired Student’s *t*-test, for non-normally distributed data analyzed by the Mann–Whitney *U* test, using GraphPad Prism version 11.0.0 for Windows, GraphPad Software, Boston, MA, USA. www.graphpad.com.

Flow cytometric analysis of spleen samples revealed distinct immunological profiles depending on the probiotic pretreatment strain administered prior to *S*Tm infection. The frequencies of Th cells were similar in both groups (Fig. 4A and B). However, frequencies of Th CD103^+^ cells within CD3^+^ and Th cells (*p* < 0.01) were lower in the EcN^G66R^ + *S*Tm group compared to the EcN^WT^ + *S*Tm group (Fig. 4D and E). Frequencies of Tc cells only within CD3^+^ T cells (*p* < 0.05) were higher in the EcN^G66R^ + *S*Tm group (Fig. 4F and G), indicating a stronger cytotoxic T cell response following infection in mice pretreated with the EcN^G66R^. Similarly, the frequencies of Tc CD103^+^ cells only within CD3^+^ cells were higher in the EcN^G66R^ + *S*Tm group compared to the EcN^WT^ + *S*Tm group (Fig. 4I). There were no changes in the frequencies of the total pool of dendritic cells or resident dendritic cells in both groups (Fig. 4K–M). In contrast, mice pretreated with the EcN^WT^ displayed significantly elevated frequencies of migratory dendritic cells within viable and CD3^−^ cells (*p* < 0.01) (Fig. 4N–P).

After the *S*Tm challenge, macrophage responses varied depending on probiotic exposure. In mice pretreated with EcN^WT^ and then challenged (EcN^WT^+*S*Tm), the frequency of splenic F4/80^+^ macrophages among viable cells was higher compared to EcN^G66R^ + *S*Tm group (*p* < 0.01)(Fig. 4Q). The M1 macrophages within viable (*p* < 0.05)(Fig. 4R) but not within F4/80^+^ cells were increased in the EcN^WT^ + *S*Tm group (Fig. 4S), however the expression of CD86 activation marker (calculated as MFI) was higher in EcN^G66R^ + *S*Tm group (*p* < 0.01)(Fig. 4V). The M2 macrophages among viable and F4/80^+^ cells were similar in both examined groups (Fig. 4T, U).

### Differential splenic immune cell profiles between EcN^WT^ and EcN^G66R^-treated mice following probiotic treatment

Given the significant differences in protection against *Salmonella* challenge observed between EcN^WT^ and EcN^G66R^-pretreated mice, including prevention of splenomegaly, reduced pathogen loads, and improved clinical outcomes with the EcN^G66R^ mutant, we sought to understand the baseline immunological differences established by these probiotic strains prior to infection. To do so, we evaluated the effects of sequential oral administration of EcN (WT and G66R) in *Salmonella*-susceptible BALB/c mice without pathogen challenge by comparing overall health, physiological features, and systemic immune profiles (Supplementary Fig. 6A). Oral administration of probiotic strains does not affect mice’s behavior, weight, or weight of internal organs, and does not cause any visible organ changes (Supplementary Fig. 6B). Bacterial enumeration from isolated systemic organs (spleen, liver, mesenteric lymph nodes) revealed no detectable CFU. Moreover, whole-body bioluminescence imaging revealed luminescent signals across animals, with no significant differences in intensity observed between EcN^WT^- and EcN^G66R^-treated animals, and signals remained confined to the gastrointestinal region with no evidence of systemic dissemination (data not shown).

To directly evaluate the relative intestinal colonization fitness of the two strains, we performed a streptomycin pretreatment competitive index experiment (Supplementary Fig. 6C). EcN^G66R^ was consistently recovered at higher levels than EcN^WT^ from fecal samples at all examined time points (geometric mean CI across all time points = 4.37). The competitive advantage of EcN^G66R^ was most prominent during the first half of the experiment (GM CI at D1 + 3h = 10.83, D2 = 8.81, D2 + 3h = 7.56, D3 = 8.06). Tissue-associated CI collected on day 5 was consistently above 1 across all examined intestinal compartments (gut CI = 2.03, intestine CI = 1.08, cecum CI = 1.85), confirming that EcN^G66R^ colonization advantage extended to the mucosal level. (Supplementary Fig 6C). 16S rRNA analysis revealed comparable *Enterobacteriaceae* levels between the EcN^WT^ and EcN^G66R^ treatment groups across multiple time points (Supplementary Table 5C), indicating that the EcN^G66R^ mutant does not alter baseline *Enterobacteriaceae* populations prior to pathogen challenge. Overall community composition was similar across treatment groups, with no major shifts in dominant families observed after administration of either probiotic strain (Supplementary Fig. 7A).

Blood ferrum concentration was significantly decreased after EcN^WT^, but not after EcN^G66R^ treatment, whereas blood glucose level was significantly decreased after treatment with both probiotic variants compared to untreated mice (Supplementary Fig. 6D). qPCR analysis of uninfected spleens showed no significant differences in IL-6, IL-10, IL-15, and IFN-γ expression between EcN^WT^ and EcN^G66R^ treatment groups (Supplementary Fig. 6E).

**Fig. 5:**
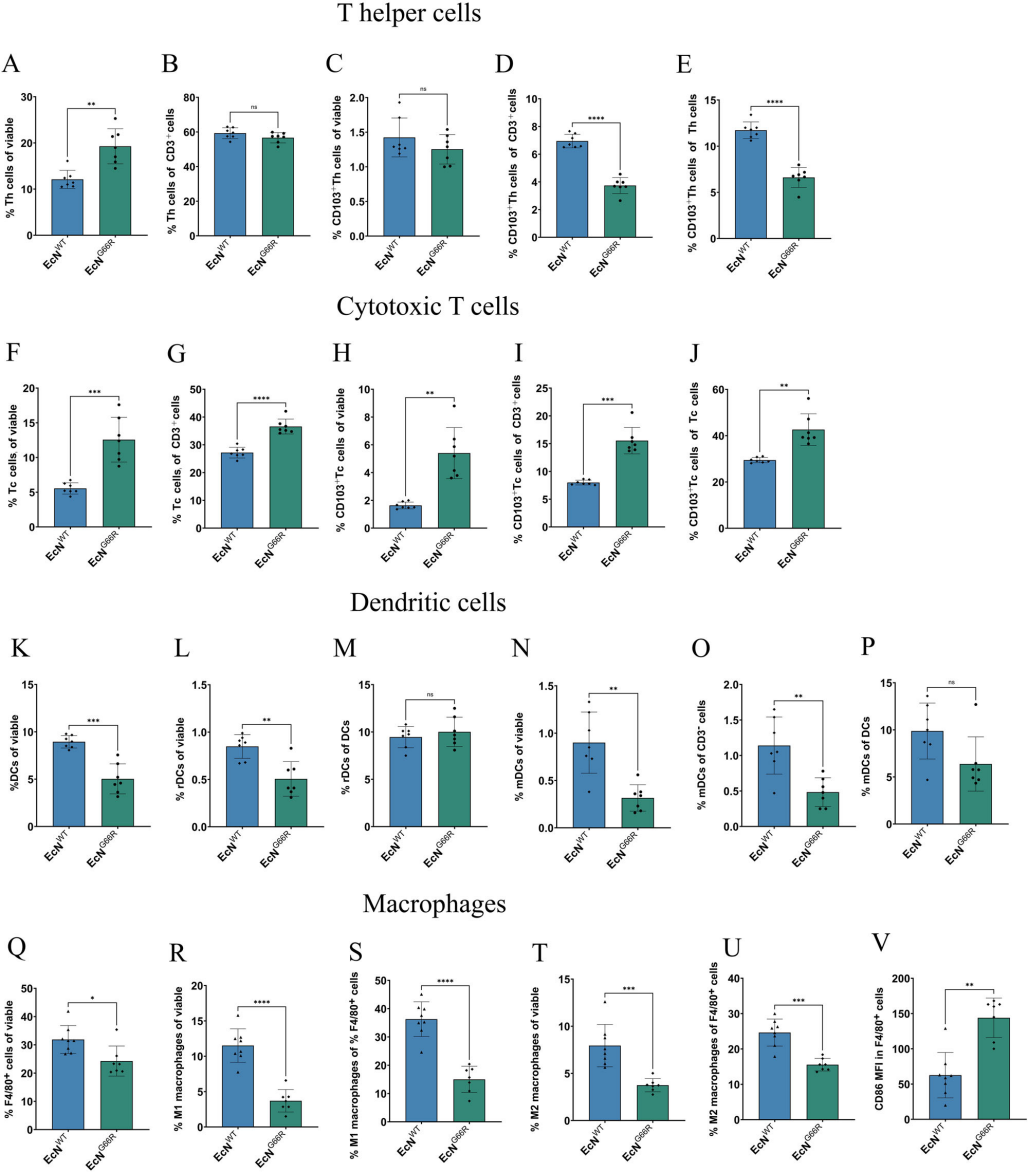
EcN^G66R^ boosts the immune response in mice*.* The data shown the percentages of: T helper (Th, CD3^+^CD4^+^) cells **A** among viable cells, **B** among CD3^+^ cells; CD103^+^ Th cells (CD3^+^CD4^+^CD103^+^), **C** among viable cells, **D** among CD3^+^ cells, **E** among Th cells; T cytotoxic (Tc, CD3^+^CD8a^+^) cells, **F** among viable cells, **G** among CD3^+^ cells; CD103^+^ Tc cells (CD3^+^CD8a^+^CD103^+^), **H** among viable cells, **I** among CD3^+^ cells, **J** among Tc cells; dendritic cells (DCs, CD3^−^CD11c^+^), **K** among viable cells; resident dendritic cells (rDCs, CD3^−^CD11c^+^CD8a^+^), **L** among viable cells, **M** among DCs; migratory dendritic cells (mDCs, CD3^−^CD11c^+^CD103^+^), **N** among viable cells, **O** among CD3^−^, **P** among DCs, **Q** F4/80^+^ macrophages (CD3^−^F4/80^+^) among viable cells; M1 macrophages (CD3^−^F4/80^+^CD86^+^), **R** among viable, **S** F4/80^+^ macrophages; M2 macrophages (CD3^−^F4/80^+^CD206^+^), **T** among viable cells, among **U** F4/80^+^ macrophages, **V** the specific fluorescence intensity of CD86 on the surface on F4/80^+^ macrophages in the mice spleens cells after treatment with EcN^WT^ (*n* = 7) and EcN^G66R^ (*n* = 7). Values are presented as mean ± SEM. Statistical significance: for normally distributed data analyzed with an unpaired Student’s *t*-test, for non-normally distributed data analyzed by the Mann–Whitney *U* test. Statistical analyses were performed using GraphPad Prism version 11.0.0 for Windows, GraphPad Software, Boston, MA, USA. www.graphpad.com.

Flow cytometric analysis of splenic leukocytes revealed distinct immune cell phenotype frequencies between EcN^WT^ and EcN^G66R^ probiotic-treated mice. The overall distribution of T cell populations differed significantly between the two groups (Fig. 5A–J). CD3^+^ lymphocytes were notably higher in EcN^G66R^-treated mice (*p* < 0.001, not shown), along with increased frequencies of Th cells within viable cells (*p* < 0.001)(Fig. 5A), indicating an increased potential for tolerogenic T-cell responses. Despite no significant differences in Th CD103^+^ cells within viable cells (Fig. 5C), there were elevated frequencies of Th CD103^+^ cells within CD3^+^ (*p* < 0.001) and Th cells (*p* < 0.01) populations in EcN^WT^ in comparison to the EcN^G66R^ group (Fig. 5D and E). Tc cells frequencies within viable and CD3^+^ cells were higher (*p* < 0.0001) in EcN^G66R^ group (Fig. 5F and G). Furthermore, the percentages of CD103^+^ Tc cells among viable (*p* < 0.0001), CD3^+^ (*p* < 0.0001), and Tc cells (*p* < 0.01) were elevated in EcN^G66R^-treated animals (*p* < 0.0001), suggesting enhanced expansion or retention of cytotoxic effector subsets (Fig. 5H–J). In contrast, EcN^WT^-treated mice exhibited a significantly higher frequency of dendritic cells within viable cells (*p* < 0.0001), along with higher frequencies of resident dendritic cells (CD11c^+^CD8^+^) within viable cells but not within CD11c^+^ cells (Fig. 5K-M). Similarly, EcN^WT^-treated mice had significantly elevated migratory dendritic cells (CD11c^+^CD103^+^) within viable (*p* < 0.01), CD3^−^ and CD11c^+^ cells (*p* < 0.01) (Fig. 5N–P). Together, these findings demonstrate that probiotic treatment caused different immune modulation in the spleen. EcN^WT^-treated mice mainly showed enhanced dendritic and regulatory T-cell activity, whereas EcN^G66R^-treated mice displayed an enrichment of effector T-cell subsets, especially cytotoxic T cells. Analysis of myeloid cells showed that EcN^WT^-treated mice also had higher levels of F4/80^+^ macrophages within viable cells (*p* < 0.05) (Fig. 5Q). Both M1 (pro-inflammatory) and M2 (anti-inflammatory) macrophage frequencies among viable cells (*p* < 0.0001 and *p* < 0.001, respectively) and F4/80^+^ cells (*p* < 0.0001 and *p* < 0.001, respectively) were present in higher frequencies in EcN^WT^-treated animals (*p* < 0.01) (Fig. 5R–U), suggesting increased macrophage recruitment. Interestingly, the CD86 MFI was again higher in the G66R group (*p* < 0.01) (Fig. 5V).

To explain the systemic immunomodulatory differences between EcN^WT^ and EcN^G66R^, we conducted a comparative RNA-seq analysis of spleens from BALB/c mice. DEG analysis showed minimal transcriptional divergence between the two strains, with only 31 genes meeting our significance criteria out of 27,604 total genes analyzed (Supplementary Table 7). Notably, this limited transcriptional divergence between EcN^WT^ and EcN^G66R^ aligns with similar minimal differences observed in our earlier in vitro MIEC cell line analysis. The EcN^G66R^ mutant-induced upregulation of 21 genes and downregulation of 10 genes compared to wild-type EcN (Supplementary Table 7 and Supplementary Fig. 6F).

The EcN^G66R^ mutant increased the expression of several inflammatory mediators that were suppressed by EcN^WT^. These included *Acod1* (aconitate decarboxylase 1), a metabolic enzyme essential for itaconate production and inflammatory macrophage polarization, as well as the acute-phase proteins *Saa3* (serum amyloid A3) and *Lcn2* (lipocalin 2). Additionally, EcN^G66R^ treatment led to higher expression of *Mmp8* (matrix metalloproteinase 8), a neutrophil-derived protease involved in tissue remodeling and inflammatory cell recruitment, and *IL-1r2* (interleukin 1 receptor type 2), a decoy receptor that regulates IL-1 signaling. The increased expression of multiple immunoglobulin variable chain genes (*Ighv1-11, Ighv14-4, Igkv2-112*) and T-cell receptor genes (*Trbv30, Trav3-1*) in the EcN^G66R^ group suggests enhanced adaptive immune activation compared to wild-type EcN. Analysis of immune-related genes revealed that only 11 genes showed significant differential expression between the two treatments, with nine upregulated and two downregulated in EcN^G66R^ compared to EcN^WT^ (*Igkv4-62, Gm8893*) (Supplementary Fig. 6G).

Overall, our results show that EcN^G66R^ serves as an immune primer and stimulates T-cell responses more effectively than EcN^WT^, resulting in better protection against *Salmonella* Typhimurium infection, as demonstrated by lower fecal shedding and decreased internal organ loads.

## Discussion

The various mechanisms of probiotic action include adhesion, competition, or immune response modulation, and modern molecular biology techniques enable genetic modifications and the enhancement of specific features to offer even more comprehensive protection^3^. *Escherichia coli Nissle 1917* (EcN) is one of the first human commensals identified for its role in protecting against enteric pathogen infections^4^, but the mechanisms and conditions behind its protective function remain unclear. In this study, we introduced three point mutations (G66R, T158P, and Y186R) into the *fimH* gene of the EcN to modify adhesive properties and examine their impact on probiotic function and protection against *Salmonella* Typhimurium (*S*Tm). All three mutations targeted specific regions of the FimH protein: G66R is located in the lectin domain near the mannose-binding pocket^17,22^, T158P is in the interdomain linker connecting the lectin and pilin domains^19^, and Y186R is found within the pilin domain^18^ (see Fig. 1A), and therefore may differentially affect the adhesin’s conformational flexibility, ligand-binding strength, and/or specificity. It has previously been shown that point mutations in the *fimH* can affect either the strength or the binding specificity to host cell structures^17–19,23,24^, not only in *E. coli* but also in various *Salmonella* serovars^25–27^, with diverse ligands identified, including β1 and α3 integrins, CD48, collagens, laminins, and fibronectin^28–30^. For example, the S62A substitution completely transforms the receptor repertoire, enabling bacterial penetration of tissue barriers and systemic dissemination^24^, while the E89K mutation redirects adhesion from yeast cells to uroepithelial cells^23^.

Despite significant differences in binding levels, the mutant strain triggers only modest transcriptional changes in vitro, primarily associated with stress response, inflammatory regulation, and cellular trafficking. Most notably, EcN^G66R^ reduced IL-6 expression compared with wild-type, suggesting that the mutant may engage alternative signaling cascades that promote tolerance rather than acute inflammation. Across multiple models, EcN consistently lowers IL-6 levels. Oral EcN reduces IL-6 and IFN-γ in acute intestinal inflammation-engineered^31^ EcN variants further suppress IL-6, TNF-α, and CXCL-1 in DSS colitis^32^, and curli-TFF3-producing EcN strains induce pronounced reductions of IL-6, IL-17A, and TNF-α in colonic tissue^33^.

EcN^G66R^ reduces *S*Tm adhesion more effectively than EcN^WT^, which aligns with the broadly described mechanism of probiotic-mediated competitive exclusion of epithelial binding sites^34–37^, decreasing pathogen load, preventing infection, and maintaining gut barrier integrity^38–40^. Beyond binding competition, *Salmonella* infections trigger pro-inflammatory responses (IL-6, IL-1β, IL-12) that drive IFN-γ production and macrophage activation^41^. In our data, both strains reduced IL-6 expression during infection compared with *S*Tm alone. This reflects strain-specific immunomodulatory strategies in which *Salmonella* typically triggers robust IL-6 and IL-8 production^42^. Differences induced by two strains align with findings that different probiotic strains employ mechanisms to suppress inflammation by reduction of IL-6, IL-8, and IL-1β, and secretion of regulatory cytokines IL-10 and IL-19^43–45^. These co-interaction experiments provided the first indication that EcN^G66R^ not only competes more effectively for physical binding sites but also programs epithelial cells to respond differently to pathogenic challenge, acting as a dual-action probiotic. Previous studies have shown that receptor-binding specificity may affect the type and strength of immune responses triggered by FimH variants. For example, the T158P mutation in adherent-invasive *E. coli* enhances binding to CEACAM6 receptors on intestinal cells. This results in increased IL-1β levels and significant intestinal inflammation in Crohn’s disease models^19^. The G66R mutation in uropathogenic *E. coli* strains activates bladder epithelial cells through CD14 and Toll-like receptors, triggers apoptosis, and attracts additional immune cells^17^. However, at this point, we cannot tell whether the enhanced epithelial cell attachment and immune modulation are directly related or act in parallel.

*Salmonella* Typhimurium is a major foodborne pathogen causing gastroenteritis and can progress to life-threatening systemic infections, particularly in vulnerable populations. BALB/c mice represent a naturally susceptible strain that develops systemic *S*Tm infection following oral administration^46^. In this model, bacteria breach the intestinal barrier and disseminate via Peyer’s patches through MLN to the spleen and liver^47^. We employed a high infectious dose to establish intestinal colonization and subsequent systemic progression, creating conditions to evaluate probiotic efficacy. In our model, in vitro adhesion of EcN^G66R^ translates to enhanced intestinal colonization in vivo, as confirmed by the streptomycin pretreatment competitive index experiment—a model that depletes resident microbiota to specifically enrich for epithelial colonizers^48^. The progressive decline in recovery of both strains, coupled with increasing inter-individual variability, may reflect the progressive reassertion of colonization resistance as the microbiota recovers from streptomycin treatment, a phenomenon well-documented for *E. coli* strains competing for locally available nutrients in the intestinal mucus layer^49,50^. It was shown before that increased adherence to the intestinal cell surface does not necessarily translate into better protection against pathogens. For example, an EcN with the envZ^P41L mutation showed up to 10-fold higher colonization of the streptomycin-treated mouse intestine, but this did not improve its effectiveness at preventing colonization by the enterohemorrhagic *E. coli* strain EDL933^50^. Our results indicate that EcN^G66R^ pre-treatment more effectively limits infection at the critical early stage of intestinal colonization (reducing shedding), thereby limiting systemic spread (lower enumeration of *S*Tm in systemic organs). The significant difference in mucosal colonization likely explains the subsequent decrease in systemic dissemination, as *Salmonella* must first establish intestinal colonization before reaching systemic sites by breaching the epithelial barrier or through M cell transcytosis^51^.

*Salmonella* transmission depends critically on fecal shedding, as superspreaders drive pathogen spread. Shedding intensity is determined by bacterial virulence factors, host genetics, and microbiota composition, and antibiotic disruption can convert low-shedders into supershedders^52^. Host genetics significantly influence these dynamics—BALB/c mice (lacking protective SLC11A1) develop acute infections with high shedding, while 129 Sv mice exhibit chronic disease^46,53^. Probiotic interventions that effectively suppress fecal shedding—as achieved with the EcN^G66R^ mutant—hold significant epidemiological value by interrupting transmission cycles in vulnerable populations.

16S rRNA sequencing data provide important ecological context for interpreting differences in protection between groups. Both probiotic strains maintained similar gut microbial communities with no major restructuring following administration, consistent with the established principle that a single introduced strain typically produces quantitative shifts in specific taxa rather than wholesale ecological reorganization^54^. Community differences were more apparent in intestinal tissue than in fecal samples, reflecting the partial correspondence between fecal and mucosa-associated microbiota^55^. The main distinction between infected groups was a marked relative expansion of *Enterobacteriaceae* in intestinal tissue of EcN^WT^ + *S*Tm mice driven by *Salmonella* mucosal colonization, which was virtually absent in EcN^G66R^ + *S*Tm animals—confirming that EcN^G66R^ limits pathogen expansion within an otherwise ecologically comparable gut environment.

Body weight loss constitutes one of the most sensitive and biologically meaningful proxies of systemic infection severity in murine salmonellosis, correlating with the extent of bacterial dissemination, cytokine-driven metabolic deterioration, and overall host physiological compromise^56^. In our model, EcN^G66R^-pretreated mice showed significantly less weight loss compared to the EcN^WT^-pretreated group, indicating a noticeably milder disease course despite identical infectious challenge. This distinction was further supported by the absence of splenomegaly in EcN^G66R^-pretreated animals—a characteristic of severe *Salmonella* infection in susceptible mouse strains^1^—alongside preserved liver and intestinal mass and lower bacterial burden across tissues. Together, these clinical and pathological parameters provide a coherent picture of reduced disease severity conferred by EcN^G66R^ pretreatment.

Blood metabolite analysis further highlights the physiological consequences of differential protection. *Salmonella* infection perturbs host glucose metabolism through increased glycolysis and pathogen-driven disruption of enteroendocrine signaling, driving systemic hypoglycemia^57,58^. Probiotics can help counteract this imbalance. In uninfected mice, both probiotic strains lowered blood glucose, whereas during *Salmonella* infection, EcN^G66R^-pretreated mice maintained significantly higher blood glucose compared to the EcN^WT^ group. This indicates that EcN^G66R^ effectively counteracts the infection-driven metabolic crash, preventing the dangerous hypoglycemia^57,58^. Moreover, EcN^WT^ treatment significantly depleted blood iron in uninfected mice, potentially reflecting siderophore-mediated iron scavenging ^9^. EcN^G66R^ did not deplete circulating iron, and this difference was no longer detectable during infection, indicating that systemic iron preservation is not the primary driver of EcN^G66R^ superior protection. The significantly higher serum magnesium levels in EcN^G66R^ + *S*Tm mice might also be relevant. At the immune level, adequate magnesium supports T helper, B cell, and macrophage responses to lymphokines^59^, whereas hypomagnesemia promotes a pro-inflammatory state via IL-6 upregulation and exacerbates systemic inflammation^60^—suggesting that its preservation may reinforce the expanded T cell populations observed in EcN^G66R^-pretreated mice while limiting excessive inflammatory damage.

EcN^G66R^ + *S*Tm mice mounted a lymphocyte-centered immune strategy characterized by CD8^+^ T cell expansion and dendritic cell recruitment, along with reduced splenic macrophage numbers, suggesting a shift toward cytotoxic immune responses that may enhance pathogen clearance. This outcome validates the concept that pathogen-specific adaptive immunity can be more effective than broad innate inflammatory responses in controlling intracellular bacteria such as *Salmonella*^61^. It was demonstrated that noncirculating tissue-resident memory T cells (both CD4^+^ and CD8^+^) in nonlymphoid tissues are vital components of immunity to *Salmonella* infection, with effective protection correlating specifically with expanded *Salmonella*-specific memory T cells that display markers of tissue residence, such as P2X7, ARTC2, LFA-1, and CD101 ^2^. The expansion of CD103+ tissue-resident Tc cells by EcN^G66R^ may serve as a mechanism for establishing long-lasting, tissue-specific immunity that cannot be achieved solely through circulating memory cells. CD11c^+^CD103^+^ dendritic cells are key in intestinal *Salmonella* infection: they are recruited from the lamina propria into the epithelium in a TLR- and chemokine-dependent manner and extend dendrites to capture luminal bacteria^62^. In *Salmonella* infection, CD103^+^ DCs help direct CD4^+^ T-cell differentiation toward immune regulation, including FoxP3^+^ Treg expansion^63,64^. Although early responses are dominated by flagellin-specific CD4^+^ T cells, CD103^+^ DCs tend to promote mucosal homeostasis^62,64^.

During *Salmonella* infection, EcN^G66R^-treated mice maintained significantly lower total macrophage frequencies compared to EcN^WT^-treated animals, yet showed higher activation states, reflected in higher expression of CD86 (calculated as MFI). This qualitative shift—fewer total macrophages but enhanced activation—may suggest more efficient immune control via adaptive mechanisms, reducing the need for massive macrophage recruitment while ensuring that the present macrophages are functionally optimized. Moreover, observed expansion of F4/80^+^ macrophages in the EcN^WT^ pretreated group explains the pathological splenomegaly characterized by macrophage accumulation in white pulp and hemophagocytic activity described in severe *Salmonella* infections^1^.

EcN^WT^-triggered response may be focused on neutrophil pathway activation, as RNA-seq results show this myeloid-dominated response, with upregulation of the core neutrophil effector program: *Ctsg* (cathepsin G), *Elane* (neutrophil elastase), *Mpo* (myeloperoxidase), and *Prtn3* (proteinase 3)—all essential components of neutrophil extracellular traps (NETs) and bacterial killing machinery^65^. The upregulation of *Cxcl2*, *Cxcl3*, *Selp* (P-selectin), *Itga2b* (integrin α2b), and multiple other factors involved in neutrophil recruitment and tissue infiltration also supports this hypothesis. In contrast, EcN^G66R^ drives extensive upregulation of diverse T-cell receptor chains (*Trav*, *Trbv* genes), co-receptors (*Cd3d*, *Cd3e*, *Cd3g*, *Cd5*, *Cd7*, *Cd28*), and coordinated expression of T-cell transcription factors (*Bcl11b, Eomes*, *Lef1*, *Bach2*, *Themis*), indicating not merely quantitative T-cell expansion but qualitative programming of effector functions. The selective upregulation of the XCL1–XCR1 chemokine axis marks a significant shift in how the immune response is directed. XCR1 is exclusively expressed on CD8α^+^ dendritic cells in mice, a specialized subset that excels at cross-presenting exogenous antigens via MHC class I molecules to prime CD8^+^ T cell responses^66,67^. This cross-presentation pathway is essential for initiating cytotoxic T lymphocyte responses against intracellular bacteria like *Salmonella* that do not directly infect antigen-presenting cells, but rather remain sequestered within phagosomes where antigens are typically restricted to MHC class II presentation. This enhanced cross-presentation may overcome a key virulence strategy of *Salmonella*: during infection with virulent *S*. Typhimurium, CD8^+^ T cell priming is considerably delayed (peaking around day 21 rather than day 7), with the phagosomal lifestyle allowing escape from host CD8^+^ T cell recognition and conferring a survival advantage to the pathogen^68^. The early immunological programming established by EcN^G66R^—with expanded XCR1^+^ dendritic cells and pre-positioned tissue-resident CD8^+^ T cells—likely accelerates this otherwise delayed priming process, eliminating the temporal window that *Salmonella* typically exploits for establishing chronic infection. By contrast, the neutrophil-recruiting CXC chemokines (*Cxcl2*, *Cxcl3*) upregulated in EcN^WT^ + *S*Tm mice lack this precision, recruiting cells with potent but non-specific antimicrobial mechanisms that inflict collateral damage while failing to establish durable adaptive immunity. The critical role of adaptive immunity in controlling even beneficial commensal bacteria is underscored by studies showing that *E. coli* Nissle can translocate and disseminate systemically in mice lacking both microbiota and adaptive immunity (germfree Rag1−/− mice, 100% mortality), while reconstitution with CD4^+^ T cells prior to challenge prevents both translocation and mortality^69^. These findings reveal two potentially distinct immunological strategies. EcN^WT^ pretreatment prompts a quick, primarily innate immune response marked by neutrophil recruitment and myeloid effector activation—a frontline defense that is strong but temporary and nonspecific. In contrast, EcN^G66R^ redirects the immune response toward adaptive mechanisms, in which early inflammatory signals activate and expand cytotoxic T cells, preparing the host for a more targeted and lasting response upon encountering a pathogen.

The enhanced protection against *Salmonella* systemic dissemination observed in EcN^G66R^-treated mice prompted us to investigate the differences among probiotic strains in the mouse model. In both groups treated with probiotics, we did not observe any changes in mouse behavior, activity patterns, or organ pathology throughout the experimental period, which is a critical safety feature for probiotic strains^3^.

In the splenic compartment under non-infected conditions, EcN^G66R^ induced a slightly more inflammatory environment than EcN^WT^, characterized by upregulation of Acod1^70^ and associated metabolic reprogramming genes. This mild inflammatory state, alongside antimicrobial effectors like Lcn2^9^ (lipocalin 2, which sequesters bacterial siderophores) and immunomodulatory molecules (Saa3, IL-1r2), suggests that EcN^G66R^ establishes a state of metabolic “readiness” or immune priming that enables rapid, effective antimicrobial responses upon pathogenic challenge^71,72^.

Infections with *Salmonella* often lead to a decline in both CD4^+^ and CD8^+^ T lymphocyte populations in spleen and systemic lymphoid tissues, impairing adaptive immune responses and enabling bacterial dissemination. By contrast, exposure or immunization with attenuated *Salmonella* frequently elicits robust expansion of antigen-specific CD4^+^ and CD8^+^ T cells and boosts host immune control^1^. Remarkably, mice treated with EcN^G66R^ showed significantly higher frequencies of CD3^+^ T cells, with increases observed in both CD4^+^ helper T cell and CD8^+^ cytotoxic T cell subsets. Notably, wild-type EcN has been previously shown to increase T helper and T cytotoxic cell frequencies in both systemic and intestinal tissues^73^. It seems that the G66R mutation may enhance adaptive immunity, which in turn limits *Salmonella* colonization and systemic spread—adding another protective layer to the reduced adhesion and competitive exclusion. This suggests that EcN^G66R^ may function as an immunological primer—similar to a live attenuated vaccine—establishing CD8^+^ T cell surveillance that enables rapid pathogen recognition and elimination upon subsequent *Salmonella* challenge, explaining the enhanced protection^22,73^. The expansion of CD8^+^ T cell populations by EcN^G66R^ is particularly significant, given that CD8^+^ T cells are critical for long-term immunity to *Salmonella*, providing both immediate cytotoxic effector functions and durable immunological memory that neutrophil responses cannot achieve. As demonstrated by vaccine studies, *Salmonella* vectors elicit robust antigen-specific CD8^+^ T cell responses with elevated IFN-γ production^22,74^. Moreover, CD8^+^ T cells have been shown in specific MHC backgrounds to accelerate *Salmonella* clearance, sometimes even more potently than CD4^+^ cells, highlighting that both T-cell arms contribute significantly to pathogen control^51^. The ability of EcN^G66R^ to expand both CD4^+^ and CD8^+^ T-cell compartments prior to pathogen exposure may equip the host with adaptive effector functions, including early IFN-γ-dependent cytotoxicity and CD4^+^-mediated sterilizing immunity, which are known to be required to limit subsequent infection^51,74,75^.

Beyond the T cell compartment, differences in the population of antigen-presenting cells were observed between EcN^G66R^- and EcN^WT^-treated mice. EcN^G66R^ treatment resulted in significantly lower total frequencies of F4/80^+^ macrophages in the spleen. *Salmonella* exploits macrophages as an intracellular replication niche^76^, where the pathogen survives within modified phagosomes called *Salmonella*-containing vacuoles (SCVs), using type III secretion systems to prevent lysosomal fusion and reprogram host cell metabolism^77^. *Salmonella* preferentially replicates within hemophagocytic macrophages, which exhibit an anti-inflammatory M2 phenotype that creates a permissive environment for bacterial survival^78^. During severe *Salmonella* infection, massive macrophage expansion and hemophagocytosis characterize pathological splenomegaly, with F4/80^+^ macrophage infiltration into white pulp contributing to tissue damage and providing bacterial sanctuaries for persistent infection^1^. The absence of this macrophage expansion in EcN^G66R^-treated mice—and the corresponding lack of splenomegaly observed in this group—may indicate the protective effect of the modified probiotic. This phenotype synergizes with the enhanced CD4^+^ and CD8^+^ T cell responses induced by EcN^G66R^, suggesting a shift toward adaptive, rather than macrophage-dominated immunity.

Collectively, we demonstrated that the G66R mutation in FimH of *E. coli* Nissle 1917 enhances the probiotic strain’s ability to protect the host against *Salmonella* infection, likely through a dual mechanism of competitive exclusion and immune priming. This leads to limited *Salmonella* intestinal colonization, lower fecal shedding, decreased systemic dissemination, and reduced infection pathology. However, to fully translate these observations into broader contexts, several limitations and avenues for future research must be addressed. While the protective phenotype of EcN^G66R^ is robust, certain mechanistic links remain correlative rather than causal: although we observed a significant expansion of T-cells, this study does not provide direct functional evidence of their absolute requirement for protection. We cannot yet formally decouple the specific contribution of these subsets from potential CD4+ help or innate immunity. The specific receptor recognized by the G66R variant has not yet been confirmed or identified. While our in vitro data support the hypothesis of expanded receptor recognition, the biochemical identity of these targets remains elusive. Currently, part of our mechanistic insights relies on RNA-Seq transcriptomics, with no additional proteomic or functional verification across all physiological compartments.

## Methods

### Bacteria, plasmids, and growth conditions

Bacterial strains and plasmids used in this study are listed in Tables 1 and 2;

**Table 2:** Plasmids used in this study

Plasmid	Characteristic	Reference
pEMG	Suicide plasmid; KmR	^80^
pEMG-G66R	Suicide plasmid; KmR; with insert carrying G66R point mutation in fimH	This study
pEMG-T158P	Suicide plasmid; KmR; with insert carrying T158P point mutation in fimH	This study
pEMG-Y186R	Suicide plasmid; KmR; with insert carrying Y186R point mutation in fimH	This study
pSW-2	Plasmid for m-toluate-inducible expression of the I-SceI enzyme; GmR	^80^
pFPV-mCherry	Plasmid contains the gene for the mCherry protein AmpR	^85^
p16Slux	Temperature-sensitive p16Slux plasmid (containing the luxABCDE operon of Photorhabdus luminescens), EryR	^82^

*Escherichia coli* Nissle 1917 (EcN), its mutants, and *Salmonella* Typhimurium SL1344 (*S*Tm) strains were grown in Luria-Bertani broth (LB). Unless otherwise specified, bacterial cultures were typically grown at 37 °C for 16 h in static conditions. *Salmonella* strains with the mCherry plasmid were grown in LB medium supplemented with 100 µg/mL of ampicillin. For animal experiments, *S*Tm was grown in LB medium supplemented with 50 µg/mL of streptomycin. Before all the experiments, *S*Tm, EcN, and its mutants were passaged three times to induce T1F production^79^.

### Mutants generation

A two-step recombineering method was used for precise genomic edits in EcN, as described earlier, with minor modifications^80,81^ (Supplementary Fig. 1). PCR was performed with Q5 High-Fidelity DNA Polymerase (New England Biolabs M0491S) for all cloning procedures according to the manufacturer’s instructions. To construct the pEMG-G66R, pEMG-T158P, pEMG-Y186R suicide plasmids (Table 2), the flanking DNA fragments surrounding the target chromosomal regions were amplified via PCR and inserted into the pEMG suicide vector between I-SceI restriction sites. The pEMG derivative was mobilized from *E. coli* S17-1λpir into EcN through conjugation. Transconjugants were selected on M9-glucose minimal medium supplemented with kanamycin (50 μg/ml). Positive EcN::pEMG transconjugant colonies were transformed with the pSW-2 plasmid and stimulated on LB agar containing gentamicin (20 μg/ml) and m-toluate (1 mM), which induced I-SceI expression. Transformants that had lost the integrated pEMG plasmid were identified based on their kanamycin-sensitive phenotype. Candidate clones were screened by replica-plating on LB agar with and without kanamycin (50 μg/mL), and PCR-verified sensitive colonies with appropriate primers. Sanger sequencing confirmed the presence of the targeted point mutations in the *fimH* gene.

### Generation of bioluminescent EcN strains

Bioluminescent EcN strains were generated using the p16Slux system as described by Riedel et al. ^82^. Plasmid p16Slux, containing the luxABCDE operon, was isolated from *E. coli* SURE cells and electroporated into erythromycin-susceptible recipient strains. Transformants were selected on LB agar with erythromycin (500 μg/ml) at 30 °C and confirmed by plasmid isolation and gel electrophoresis. Chromosomal integration of the lux operon was induced by cultivating transformants at 42 °C for 24 h under erythromycin selection. Integration was verified by colony PCR using primers 16S_rev_XhoI (CTGATCTCGAGGGCGGTGTGTACAAGG)^82^ and 16S_fwd_int (ATTAGCTAGTAGGTGGGGTAACGGCTCACCTAGG)^82^, which target the expected 1150 bp fragment, resulting in a 1150 bp amplicon. The bioluminescence of positive integrants was measured using a SPARK Plate Reader (TECAN, Switzerland) with serial dilutions of overnight cultures, and strains with comparable luminescence levels were selected.

### Generation of nalidixic acid-resistant strains

Nalidixic acid-resistant mutants of EcN^WT^ and EcN^G66R^ were created through direct selection on solid medium. Overnight cultures of each strain were plated (50–100 µl) onto LB agar supplemented with nalidixic acid at increasing concentrations (10, 25, 50, 75, 100, and 200 µg/ml) and incubated for 20 h at 37 °C. Colonies that appeared on plates containing 25–75 µg/ml nalidixic acid were re-streaked onto LB agar with 50 µg/ml nalidixic acid to verify the stability of the resistance phenotype.

### Cell cultures and preparation of the co-cultures

Porcine intestinal epithelial cell line IPEC-J2 was cultured in Dulbecco’s modified Eagle’s medium (DMEM) (Biowest, France) supplemented with 10% fetal bovine serum (FBS; Biowest), 2 mM glutamine, and antibiotics (penicillin, streptomycin; Biowest). The mouse intestinal epithelial cell line (MIEC) was grown in muINTEPI Medium (InSCREENeX, Germany). Both at 37 °C with 5% CO_2_. All cell lines were either passaged twice a week or had the medium replaced every other day. For all experiments, epithelial cells were seeded in 24-well or 6-well plates at a density of 2 × 10^5^ cells or 1 × 10^6^ per well the day before the assay and used when monolayers were established.

### Probiotic adhesion and competitive exclusion assay

Adhesion assays were prepared as previously described^83^ with minor modifications. Cell monolayers were prepared as described above, washed with PBS, and 400 or 800 μl of DMEM without FBS or antibiotics was added to each well. For probiotic adhesion and MOI-dependent adhesion assays: EcN^WT^ and EcN mutants were adjusted to MOI 10, 100 and 1000 and added to the appropriate wells. DMEM alone served as a control. The plates were incubated for 2 h at 37 °C and processed as described below. For competitive exclusion assays, after the initial incubation, cells were washed with PBS to remove non-attached probiotic bacteria. Subsequently, 400 μL of DMEM and 100 μL of *S*Tm suspension (MOI 100) were added, followed by a further 2-h incubation at 37 °C. Afterwards, cells were washed three times with PBS and prepared for further analysis. For adhesion quantification, PBS was removed from each well, and 200 μL of sterile 1% PBST was added. The plates were incubated for 10 min at room temperature on a rocking shaker. Then, 800 μL of PBS was added to each well, and the cells were evenly suspended throughout the entire volume. Serial dilutions were prepared in 96-well plates and plated on LB agar plates with or without 100 μg/mL ampicillin, followed by overnight incubation at 37 °C. Ampicillin selection was used to distinguish *S*Tm from EcN in competitive exclusion assays. Colony counts were performed, and CFU/mL were calculated for each sample and strain.

### Total RNA isolation and qPCR

Total RNA was extracted from the MIEC cell line after co-culture with EcN^WT^ and its mutants using TRIzol™ Reagent (Invitrogen™, Thermo Fisher Scientific, USA) following the manufacturer’s protocol. The concentration and purity (*A*_260_/*A*_280_ ratio) were assessed using a NanoDrop 2000c (Thermo Fisher Scientific). Total RNA (2 µg per reaction) was reverse transcribed into complementary DNA (cDNA) using the High-Capacity cDNA Reverse Transcription Kit (Thermo Fisher Scientific), following the manufacturer’s instructions. The resulting cDNA served as a template for quantitative PCR (qPCR), performed on a QuantStudio™ 5 Real-Time PCR System (Applied Biosystems, USA) using EvaGreen dye (Biotium, USA), amplification conditions as follows: initial denaturation at 95 °C for 3 min, followed by 35 cycles of denaturation at 95 °C for 15 s, annealing at 56 °C for 25 s, and extension at 72 °C for 30 s. A melt curve analysis was performed over a temperature range of 60–95 °C, with appropriate step settings, to verify product specificity. All reactions were conducted in technical triplicate, with at least three biological replicates per condition. Relative gene expression levels were calculated using the comparative ΔΔCt method and expressed as relative quantification (RQ) values. Gene expression was normalized to the *Actb* housekeeping gene and calibrated to untreated MIEC cells or spleen tissue from uninfected mice. Primer sequences are provided in Supplementary Table 2.

### Total RNA-sequencing

Total RNA was extracted as described above. RNA integrity and potential contamination were evaluated with the Multina system (microchip electrophoresis system for DNA/RNA analysis MCE™-202 MultiNA)(SHIMADZU, Japan). RNA samples from three independent biological replicates per condition (both cell culture and tissue samples) were pooled and subsequently submitted to Novogene for transcriptome sequencing. Upon receipt, all RNA samples underwent additional quality control assessments by Novogene to confirm RNA integrity and purity prior to library preparation and sequencing.

### RNA sequencing data analysis

RNA-seq data were analyzed in R (2025.05.1) in RStudio using the packages readxl, openxlsx, dplyr, ggplot2, stringr, tidyr, pheatmap, RColorBrewer, tibble and ggrepel—custom functions for filtering, categorization, and identification of differential genes (DEGs). Comprehensive summaries were generated to quantify the number of expressed genes, significantly regulated genes, and genes meeting combined TPM, fold-change, and padj criteria. Raw reads were converted to TPM, and genes with TPM > 1 in both samples were retained for further analysis. Unique genes were defined as those with high expression in one sample (>5 TPM) and low expression in the other (<2 TPM). DEGs were identified at |log₂FC| thresholds > 1 and padj < 0.05, classifying them as up- or down-regulated. Genes that did not meet the thresholds were considered nonessential. Immune-related genes were identified by matching DEGs with GO annotations and KEGG pathways (29 files), using keywords and GO terms related to the regulation and activation of the immune response. For better characterization of expression changes, a difference score (range of changes × max |log₂FC|) was calculated for each gene, including only genes with TPM > 1 and padj < 0.05. The 50 genes with the highest scores were selected and plotted in a scatter plot with log₂FC = 0 and ±1 threshold lines, ordered by mean absolute expression change.

### In vivo experiments

The animal experimental procedures were approved by the Local Ethics Committee for Animal Experimentation in Wrocław (Resolution No. 060/2022/P1 dated January 18, 2023). Female BALB/c mice (5–6 weeks old) were obtained from Anlab (Prague, Czech Republic). Throughout the experiment, animals were maintained under controlled environmental conditions. The mice underwent a 5-day acclimation period followed by gentle handling sessions prior to experimental procedures. Mice were randomly assigned to four different groups: EcN^WT^ + PBS, EcN^WT^ + *S*Tm, EcN^G66R^ + PBS, and EcN^G66R^ + *S*Tm. All four groups received the appropriate probiotic bacterial suspension (EcN^WT^ or EcN^G66R^) at 10⁸ CFU in 0.1 ml PBS via intragastric gavage using a stainless steel ball-tipped gavage needle. This treatment was repeated daily for 5 consecutive days at 24-h intervals. To monitor the colonization and persistence of the probiotic, fecal samples were collected on days 1 and 5 of probiotic administration, homogenized in PBS, serially diluted, and plated on LB, LB with streptomycin, and MacConkey Agar to count viable bacteria. On the 6th day, feed and water were withheld for 2 h for all animals before oral administration of a single infectious dose of *S*Tm of 10⁸ CFU in 0.1 mL PBS via intragastric gavage to the EcN^WT^ + *S*Tm, EcN^G66R^ + *S*Tm groups and 0.1 mL of PBS to EcN^WT^ + PBS, EcN^G66R^ + PBS groups. Animals were monitored daily for clinical signs, including coat condition, locomotor activity, posture, and changes in feeding and drinking behavior. Body weights were recorded daily throughout the experiment, and fecal samples were collected for further analysis. After infection, mice were monitored using the Lago X bioluminescence imaging system (Spectral Instruments Imaging, USA) with Aura Imaging Software v.4.0 to track luminescent bacterial signals. For whole-body imaging, mice were anesthetized with 5% isoflurane and maintained under anesthesia during image acquisition. Mice were euthanized 6 days after *S*Tm infection, and their spleens, livers, lymph nodes (MLN), gallbladders, small and large intestines, were collected and weighed. Blood was collected at necropsy for further analysis. Tissue samples were homogenized in 1 ml of PBS, diluted, and plated on LB supplemented with streptomycin to determine bacterial loads. Additionally, spleen tissues were immediately placed in RNAlater solution (Thermo Fisher Scientific) and stored at −80 °C until further processing. Collected feces were homogenized and plated daily after infection as described above.

To directly compare the intestinal colonization of EcN^WT^ and EcN^G66R^, antibiotic-marked strains were used: EcN^WT^ and EcN^G66R^ carrying an erythromycin resistance marker (EcN^WT^-EryR, EcN^G66R^-EryR) and EcN^WT^ and EcN^G66R^ carrying nalidixic acid resistance (EcN^WT^-NalR, EcN^G66R^-NalR). Mice received a single oral dose of streptomycin (20 mg per mouse in 0.1 ml PBS) via intragastric gavage 24 h before starting probiotic treatment. Mice were randomly divided into two groups (4 mice each) and given a mixed probiotic suspension daily for 5 days, as described above: Group 1 received EcN^WT^-EryR and EcN^G66R^-NalR; Group 2 received EcN^G66R^-EryR and EcN^WT^-NalR (5 × 10⁸ CFU of each strain; total of 10⁹ CFU per mouse in 0.1 ml PBS). Fecal samples were collected twice daily—at 3 and 24 h post-administration. On day 5, at the study endpoint, mice were euthanized, and the small intestine and cecum were excised and processed as described above. Fecal and tissue homogenates were cultured on LB agar supplemented with erythromycin (100 µg/ml) or nalidixic acid (100 µg/ml) for differential enumeration. The competitive index (CI) was calculated for each mouse at each time point using the formula: CI = (EcN^G66R^_out/EcN^WT^_out)/(EcN^G66R^_in/EcN^WT^_in), where CFU recovered from fecal samples is divided by CFU in the administered inoculum. A CI > 1 indicates a colonization advantage for EcN^G66R^; a CI < 1 indicates the opposite. Samples with zero CFU for either strain were excluded, as well as one extreme outlier (CI > 500). CI values from both groups were combined for analysis. The geometric mean CI was calculated at each time point and across all time points.

### Splenocyte isolation

Spleens harvested from BALB/c mice and mechanically disrupted using sterile syringe plungers through 40 μm cell strainers (BD Falcon, USA) in 2 ml of red blood cell lysis solution (0.83% NH₄Cl), centrifuged (300×*g*, 10 min, 4 °C), and washed twice with a sorting buffer (PBS, 2% FBS, and 2 mM EDTA). Cells were cryopreserved in RPMI-1640 medium (Biowest) supplemented with L-glutamine, penicillin/streptomycin, HEPES, and 10% FBS, with 50% FBS and 10% DMSO at −80 °C.

### Flow cytometry

Cells were rapidly thawed at 37 °C with gradual addition of warm RPMI-1640 medium, centrifuged (300×*g*, 5 min, RT), washed with fresh medium (200×*g*, 10 min, RT), and PBS (300×*g*, 5 min, RT). Cell concentration was adjusted to 1 × 10⁶ cells per 100 μl PBS and stained for 15 min, RT with Zombie Green (BioLegend, USA) to assess viability and blocked for 10 min, RT for non-specific binding with Mouse FcX (BioLegend). Then the cells were incubated for 20 min, 4 °C, with antibodies listed in Supplementary Table 3 and appropriate isotype controls. After two washes with FACS buffer (PBS with 0.5 mM EDTA, 0.002% sodium azide, 1% FBS), cells were fixed with 1% fixation buffer (Biolegend) (20 min, 4 °C). Following two additional washes, samples were analyzed by flow cytometry on the BD FACSLyric™ Clinical Flow Cytometry System (Becton Dickinson), acquiring at least 200,000 events per sample at a flow rate of 600-80 events. Cytometer Setup and Tracking beads (CS&T Research Beads, Becton Dickinson) and compensation beads (BD™ FC Beads 7-Color Kit) were used for automated quality assurance, compensation adjustments, and control of machine performance. Data were analyzed using FlowJo™ software version 10.8.1 (Becton Dickinson), acquiring at least 200,000 events per sample. The percentage of T helper (CD3^+^CD4^+^), T cytotoxic (CD3^+^CD8^+^), M1 macrophages (CD3^−^F4/80^+^CD86^+^), M2 macrophages (CD3^−^F480^+^CD206^+^), resident (CD3^−^CD11c^+^CD8^+^), and migratory (CD3^−^CD11c^+^CD103^+^) dendritic cells (CD3^−^CD11c^+^) was calculated as the total number of viable cells, CD3^+^ cells, or the parent population during gating. The relative levels of CD86 in F4/80^+^ macrophages were calculated as follows: median fluorescence of cells stained with anti-CD86 antibody minus median fluorescence of cells stained with isotype-matched control (Supplementary Table 3). The gating strategy is presented in Supplementary Fig. 2A and B.

### 16s rRNA gene sequencing

For 16S rRNA gene sequencing, samples were collected across various experimental groups and time points to evaluate bacterial community composition during probiotic colonization and after *Salmonella* infection. For the probiotic colonization study (uninfected mice, fecal samples were collected at three time points: day 5 of probiotic administration (D5), day 6 from the first administration, and one day after stopping probiotic treatment (D6), and day 10 from the first administration (D10) from both EcN^WT^ and EcN^G66R^ treatment groups. For the *Salmonella* challenge experiment, fecal samples were collected at day 5 of probiotic administration before pathogen challenge (D5), day 1 *pi* (D1), and day 4 *pi* from both EcN^WT^ + *S*Tm and EcN^G66R^ + *S*Tm groups (D4). Additionally, intestinal tissue samples were collected during necropsy at day 6 post-infection from infected mice (EcN^WT^ + *S*Tm and EcN^G66R^ + *S*Tm) and at the equivalent time point from uninfected probiotic-treated control mice (EcN^WT^ and EcN^G66R^). For each experimental group and time point, individual samples from mice within the same treatment group were pooled and sent to GenXone S.A. for DNA extraction, 16S rRNA gene library preparation, and sequencing.

### Mutation visualization

Predicted 3D structures of the *Escherichia coli* Nissle 1917 FimH protein (wild-type and single-point mutants G66R, T158P, and Y186R) were generated using ColabFold (implementation of AlphaFold2). The obtained PDB models were visualized, annotated, and rendered in UCSF ChimeraX^84^. Mutated residues were highlighted and labeled, and domain coloring was applied to distinguish the receptor-binding and pilin domains. High-resolution images were rendered using the ray-tracing option in ChimeraX for figure preparation.

### Statistical analysis

Statistical analyses were performed using GraphPad Prism version 11.0.0 (GraphPad Software, San Diego, CA, USA). Specific statistical tests used for each experiment are indicated in the figure legends. *P*-values < 0.05 were considered statistically significant and are indicated as follows: *p < 0.05, **p < 0.01, ***p < 0.001, ****p < 0.0001.

## Supplementary information

Supplementary Information

## Data Availability

Further information and requests for resources and reagents should be directed to and will be fulfilled by the Lead Contact, Krzysztof Grzymajło (krzysztof.grzymajlo@upwr.edu.pl).

## Materials availability

Plasmids and bacterial strains generated in this study are available upon request to the Lead Contact.

## Code availability

RNA-Seq and 16S sequencing data are available in the RepOD repository (10.18150/CVTMAT) and will be released in September 2026. All other data supporting the findings of this study are available from the corresponding author upon reasonable request.
