# Supplementary material for: Genetically engineered probiotic E. coli Nissle 1917 enhances protection against Salmonella via increased adhesion and systemic T-cell responses
Source: NPJ Biofilms Microbiomes. 2026 May 21;12:121. doi: 10.1038/s41522-026-01011-w (PMC13275912; doi:10.1038/s41522-026-01011-w)
Supplement: Supplementary file 1 — Supplementary Information [file 41522_2026_1011_MOESM1_ESM.pdf]

SUPPLEMENTARY INFORMATION

Supplementary Figure 1

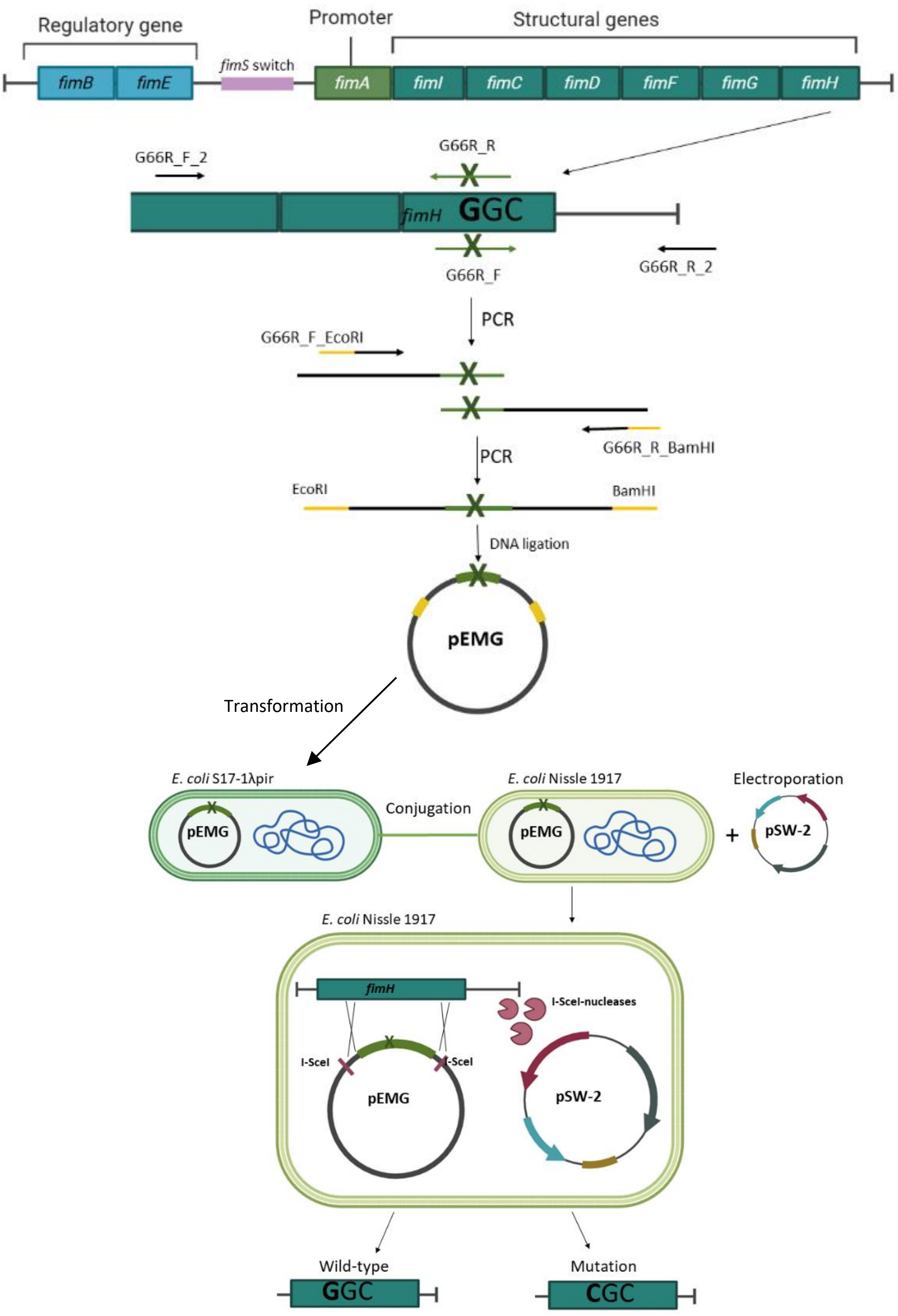

Supplementary Figure 1: Schematic of point mutation generation in *Escherichia coli* Nissle 1917, using the G66R mutation as an example. Two sets of PCR reactions with mutagenic and external primers generated overlapping fragments, which were joined by overlap extension PCR to form a full-length fragment containing the desired mutation, flanked by EcoRI and BamHI restriction sites. The mutated fragment was cloned into the pPMG plasmid between I-SceI sites. The pEMG derivative was mobilized from *E. coli* S17-1λpir to EcN by conjugation, and transconjugants were selected. EcN::pEMG colonies were then transformed with pSW-2 and plated on medium with gentamicin and m-toluate to induce I-SceI. Induced cleavage of the integrated pEMG triggered recombination that resolved the plasmid and introduced the desired mutation.

Supplementary Figure 2A

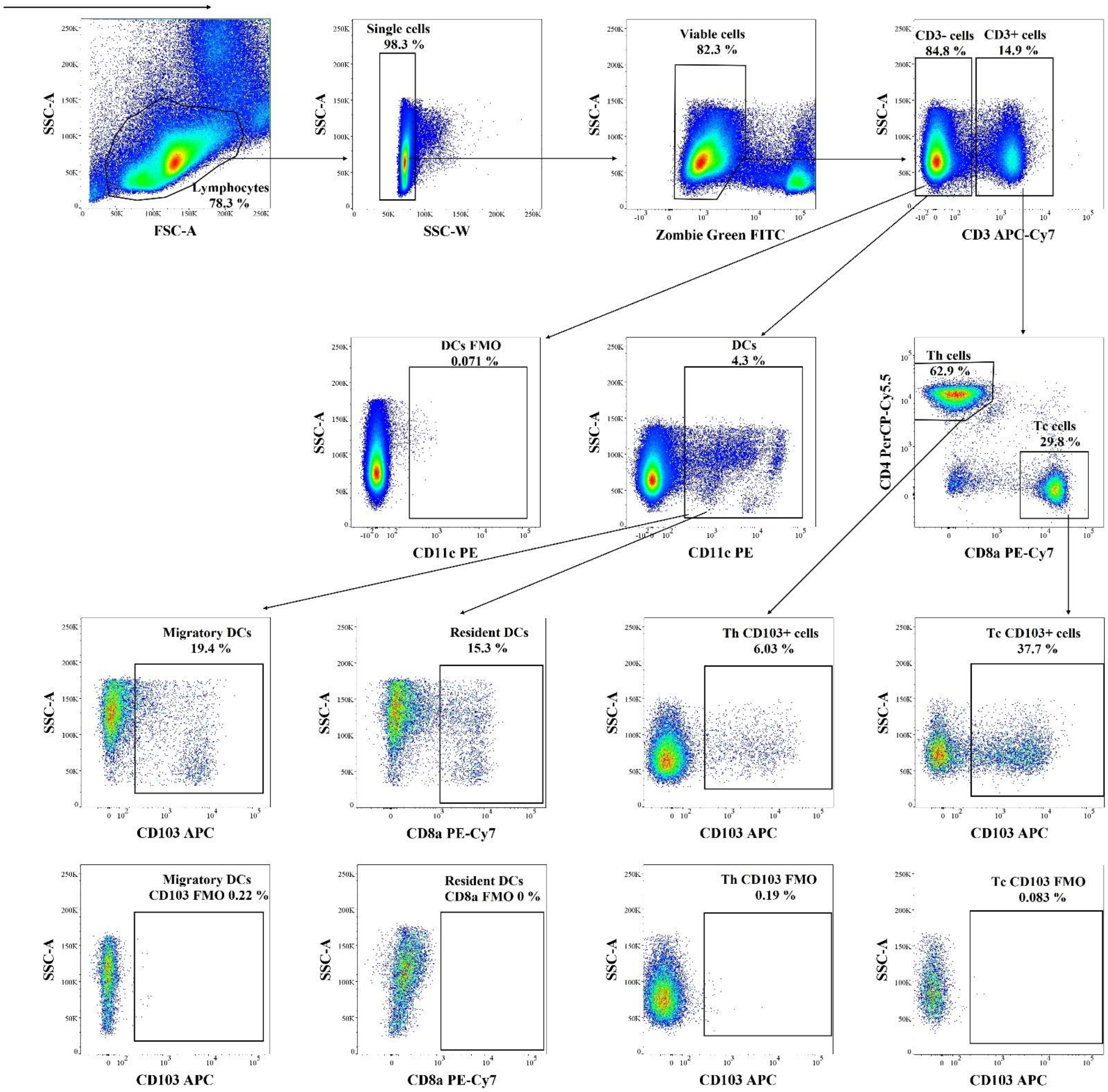

Supplementary Figure 2A Representative dot plots for the process of gating of T helper (Th, $CD3^+CD4^+$) cells, $CD103^+$ Th cells ($CD3^+CD4^+CD103^+$), T cytotoxic (Tc, $CD3^+CD8a^+$) cells, $CD103^+$ Tc cells ($CD3^+CD8a^+CD103^+$), resident dendritic cells (rDCs, $CD3^-CD11c^+CD8a^+$) and migratory dendritic cells (mDCs, $CD3^-CD11c^+CD103^+$) in spleens of mice.

Supplementary Figure 2B

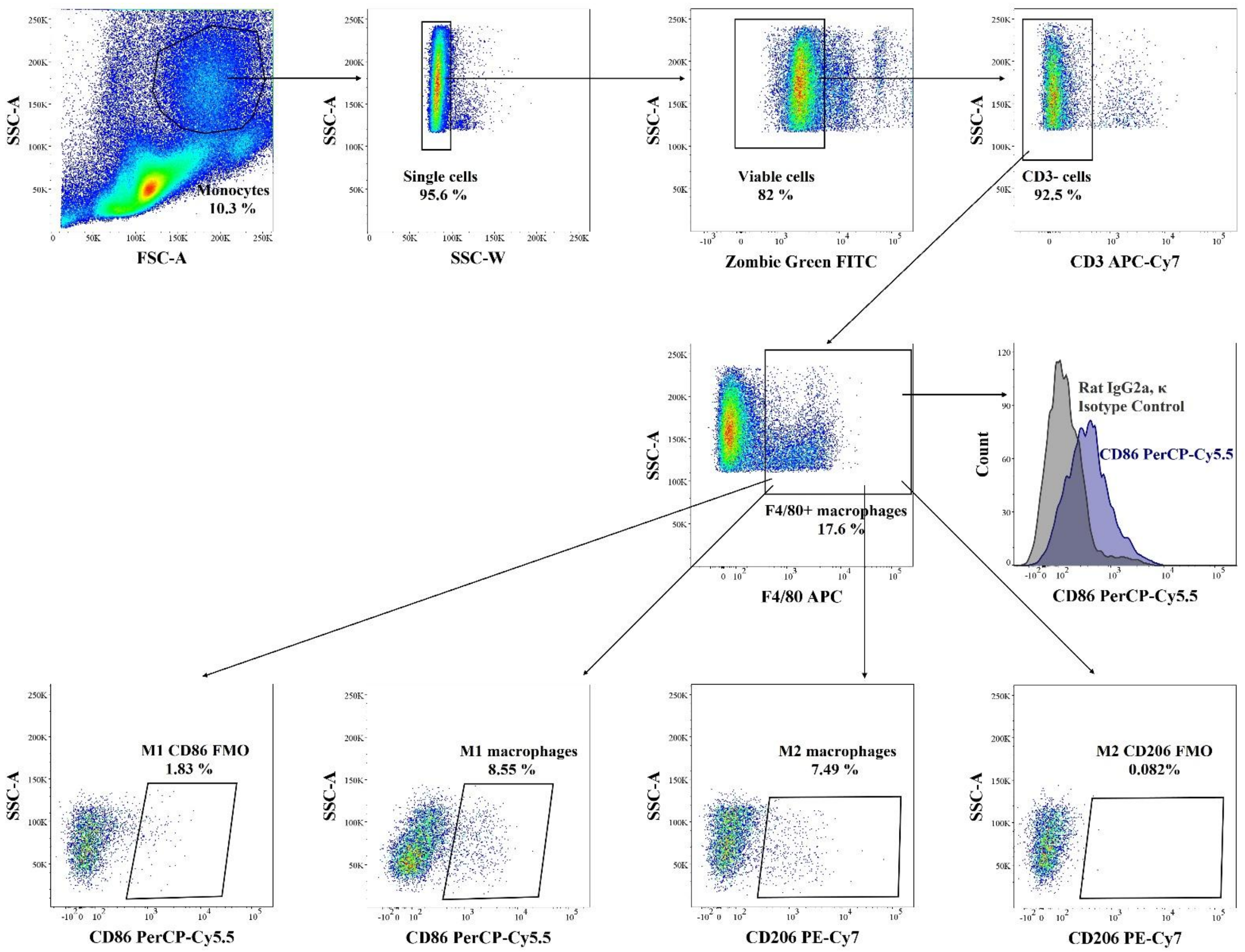

Supplementary Figure 2B Representative dot plots for the process of gating of F4/80$^+$ macrophages, M1 macrophages (CD3$^-$F4/80$^+$CD86$^+$), M2 macrophages (CD3$^-$F4/80$^+$CD206$^+$) and representative histogram of CD86 expression (blue histogram) overlaid with those in the respective isotype-matched controls (grey histogram) in spleens of mice.

Supplementary Figure 3A

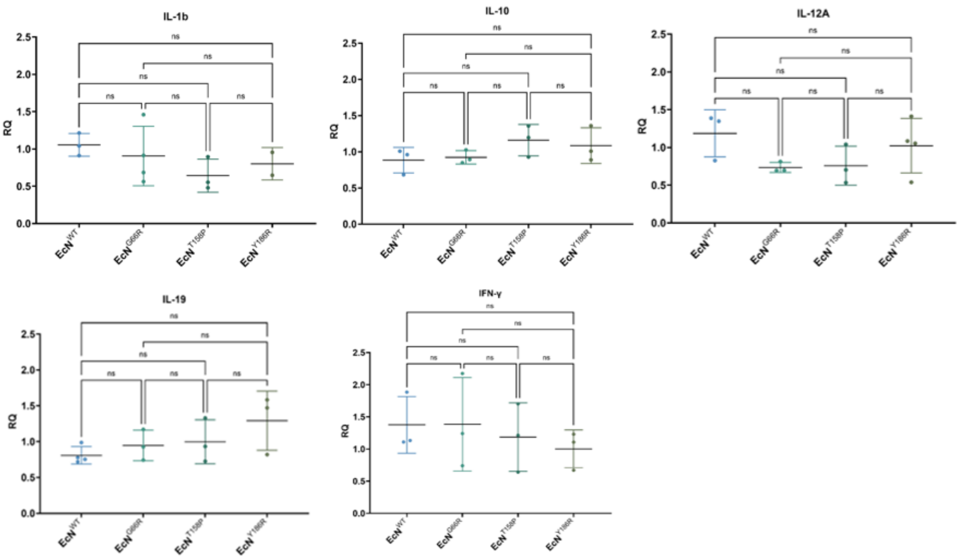

Supplementary Figure 3A: Relative expression of *IL-1β, IL-10, IL-12, IL-19*, and *IFN-γ* following incubation of the MIEC with either EcN^{WT} (blue) or EcN^{G66R} mutant (green). No statistically significant differences were observed. Statistical analysis was conducted using one-way ANOVA with Tukey's post-hoc test (for *IL-10, IL-19*) or Kruskal-Wallis test with Dunn's post-hoc test (for *IL-12, IL-1β, IFN-γ*).

Supplementary Figure 3B

A

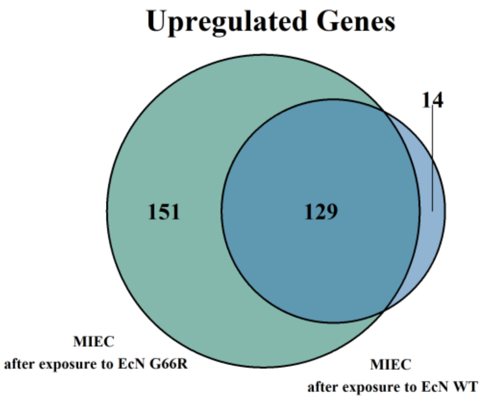

B

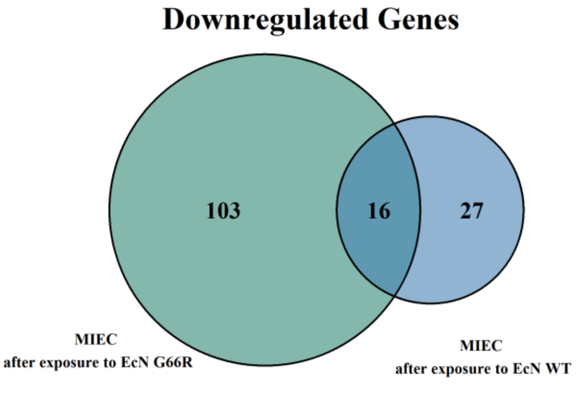

Supplementary Figure 3B: Venn diagram showing upregulated (A) and downregulated (B) genes from MIEC incubated with either EcN^{WT} (blue) or EcN^{G66R} mutant (green), in comparison to the untreated cell line. The overlapping regions represent genes shared by the compared groups. Selection criteria: |log2FC| > 1; adjusted P value < 0.05; TPM > 1 in both conditions.

Supplementary Figure 3C

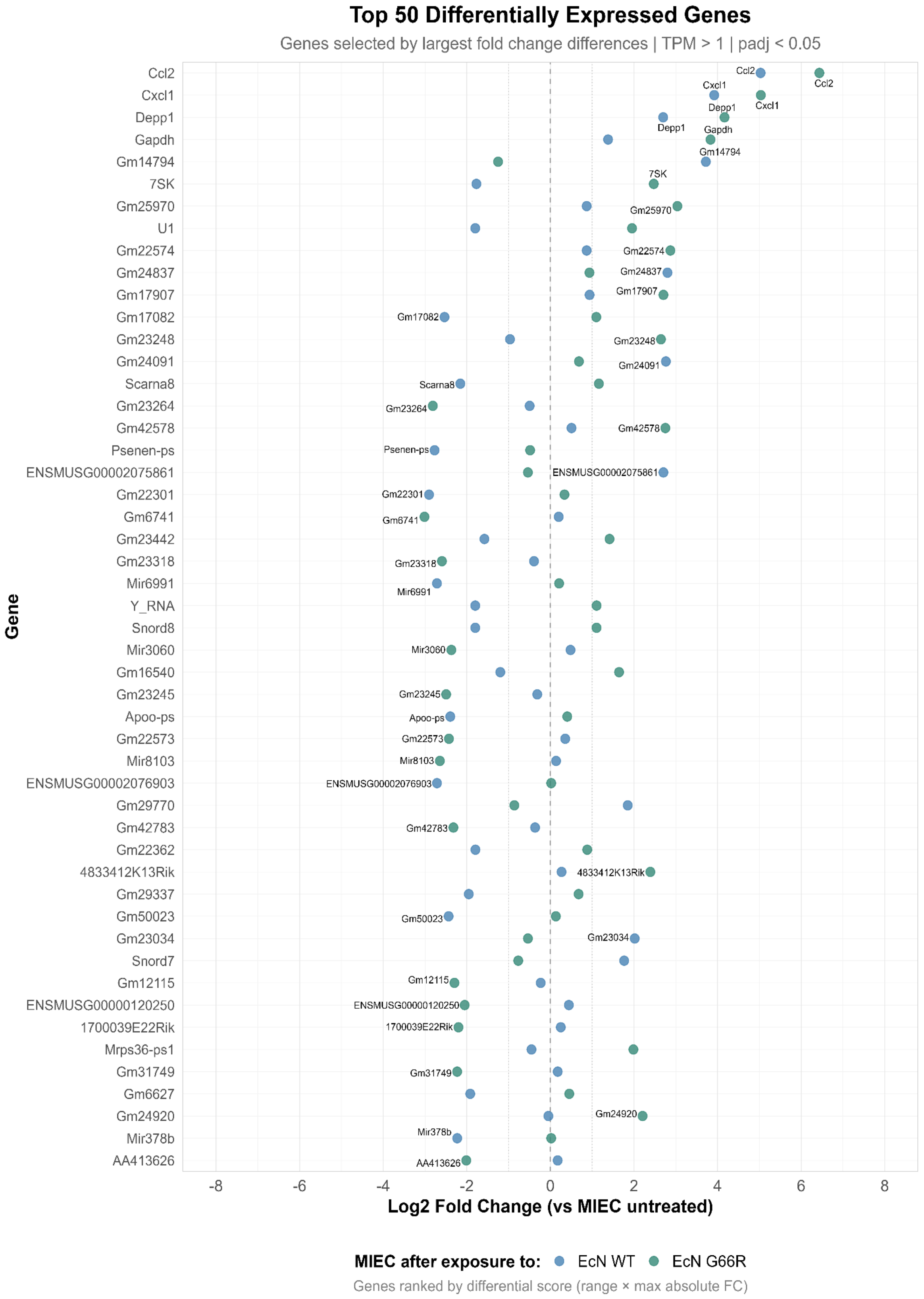

Supplementary Figure 3C: Top 50 differentially expressed genes in MIEC following exposure to EcN^{WT} and EcN^{G66R}. Dot plot showing log2 fold changes (relative to untreated MIEC) for the 50 genes with the greatest differential expression between EcN^{WT} (blue) and EcN^{G66R} (green) treatments. Genes were selected based on a differential score calculated as the product of the fold change range and the maximum absolute fold change between the two conditions. Only genes with TPM > 1 in both treatment and control conditions and adjusted p-value < 0.05 were included. Vertical dashed line indicates no change (log2FC = 0); dotted lines mark the ±1 log2 fold change threshold. Genes are ranked by mean absolute fold change across conditions.

Supplementary Figure 4A

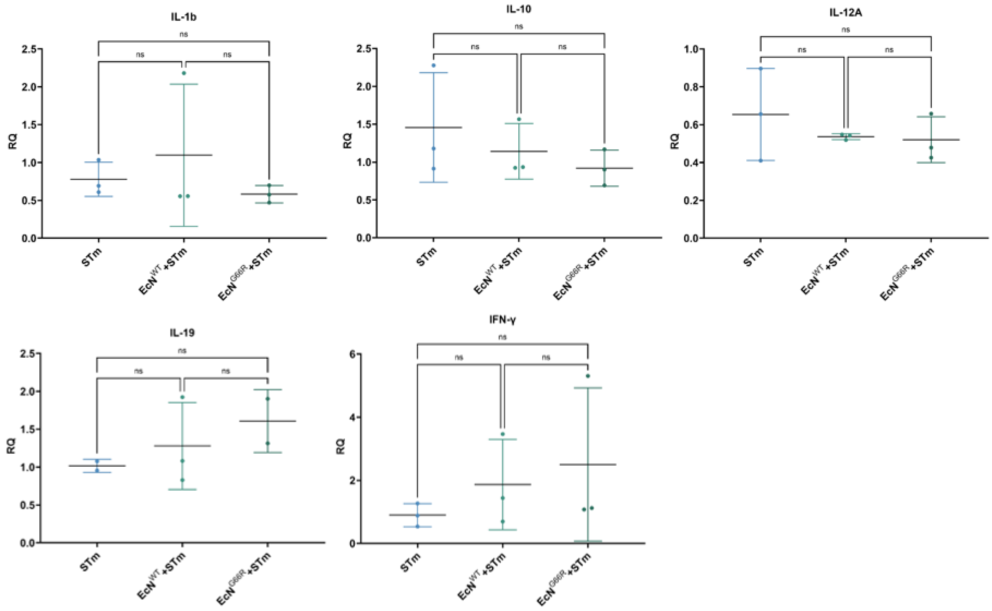

Supplementary Figure 4A: Relative expression of *IL-1β, IL-10, IL-12, IL-19*, and *IFN-γ* following incubation of the MIEC with EcN^{WT} + *S*Tm, EcN^{G66R} + *S*Tm, or *S*Tm alone. No statistically significant differences were observed (ordinary one-way ANOVA).

Supplementary Figure 4B

A

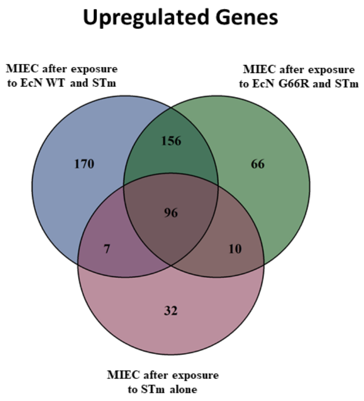

B

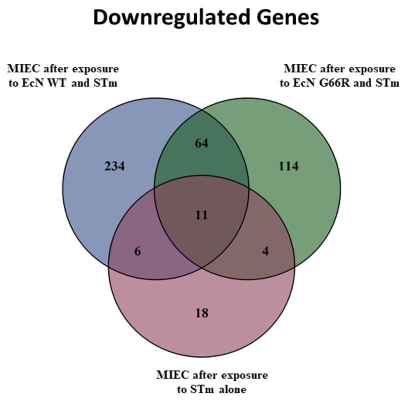

Supplementary Figure 4B: Venn diagram for upregulated (A) and downregulated (B) genes after MIEC incubated with EcN^{WT} and STm (blue), EcN^{G66R} and STm (green) or STm alone (purple) compared to the untreated cell line. The overlapping regions represent genes shared by the compared groups. Selection criteria: |log2FC| > 1; adjusted P value < 0.05; TPM > 1 in both conditions.

Supplementary Figure 4C

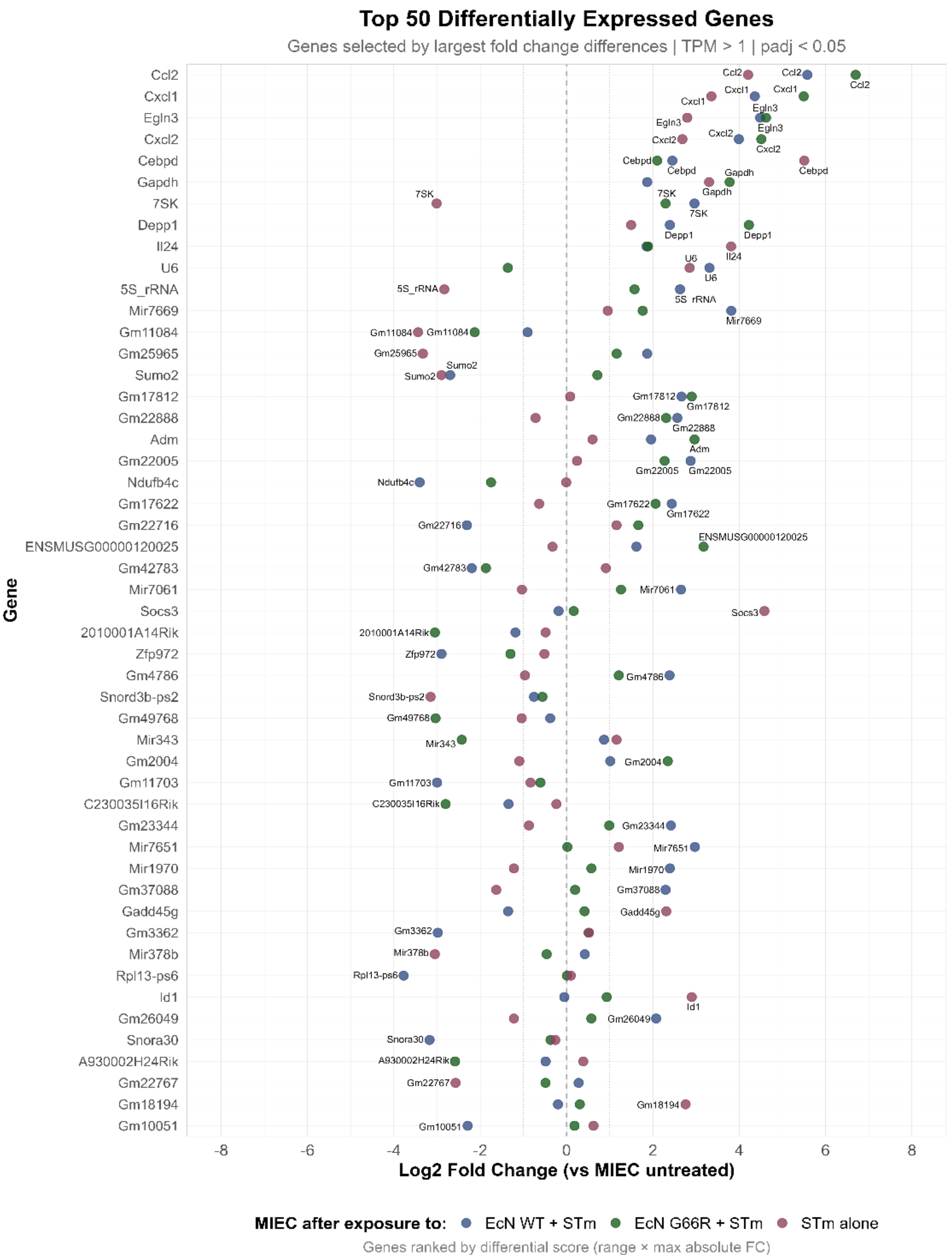

Supplementary Figure 4C: Top 50 differentially expressed genes in MIEC following exposure to STm with or without probiotic pretreatment**.** Dot plot showing log2 fold changes (relative to untreated MIEC) for the 50 genes with the greatest differential expression across three conditions: EcN^{T} + STm (blue), EcN^{66R} + STm (green), and STm alone (purple). Genes were selected based on a differential score calculated as the product of fold change range and the maximum absolute fold change between the two conditions. Only genes with TPM > 1 in both treatment and control conditions and adjusted p-value < 0.05 were included. Vertical dashed line indicates no change (log2FC = 0); dotted lines mark the ±1 log2 fold change threshold. Genes are ranked by mean absolute fold change across conditions.

Supplementary Figure 5A

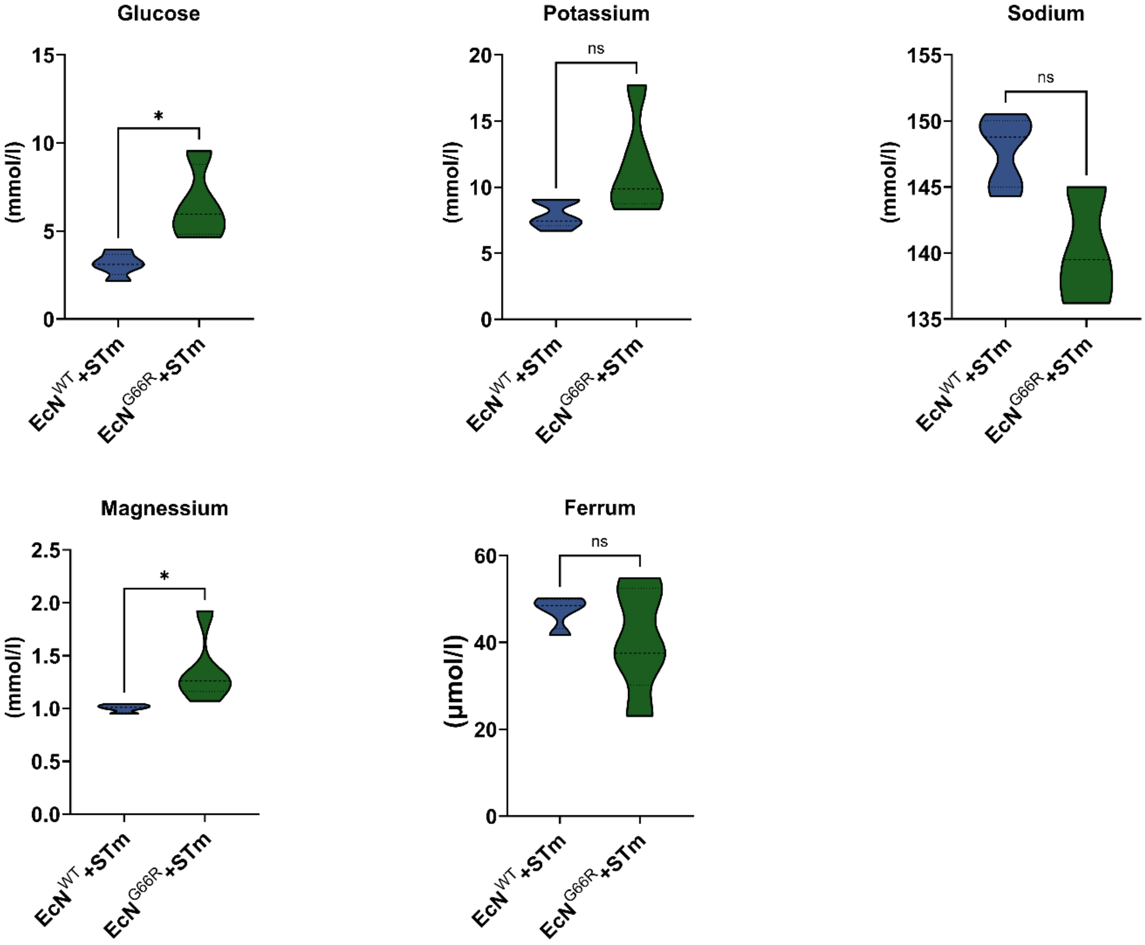

Supplementary Figure 5A. Blood parameter levels in BALB/c mice following STm infection after probiotic pre-treatment are depicted as violin plots showing serum concentrations of selected biochemical markers in mice pre-treated with either EcN WT (blue) or EcN G66R mutant (green). Statistical analysis performed using unpaired Welch's test (glucose, potassium, sodium, ferrum) or unpaired t-test or Mann-Whitney test (magnesium).

Supplementary Figure 5B

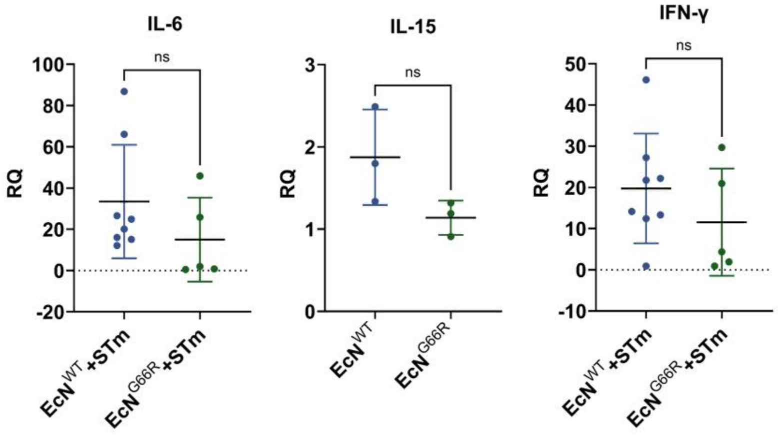

Supplementary Figure 5B. Cytokine gene expression in spleens of BALB/c mice following probiotic pretreatment and STm infection - EcN^{WT} (blue) or EcN^{G66R} mutant (green). Each data point represents an individual mouse, and horizontal lines indicate the mean ± SEM. Statistical analysis performed using the unpaired Welch's test.

Supplementary Figure 6A

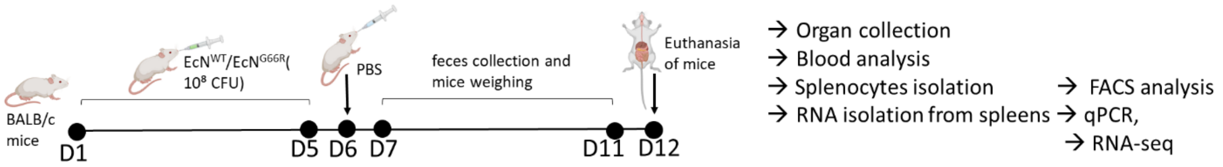

Supplementary Figure 6A Schematic representation of the animal experiment

Supplementary Figure 6B

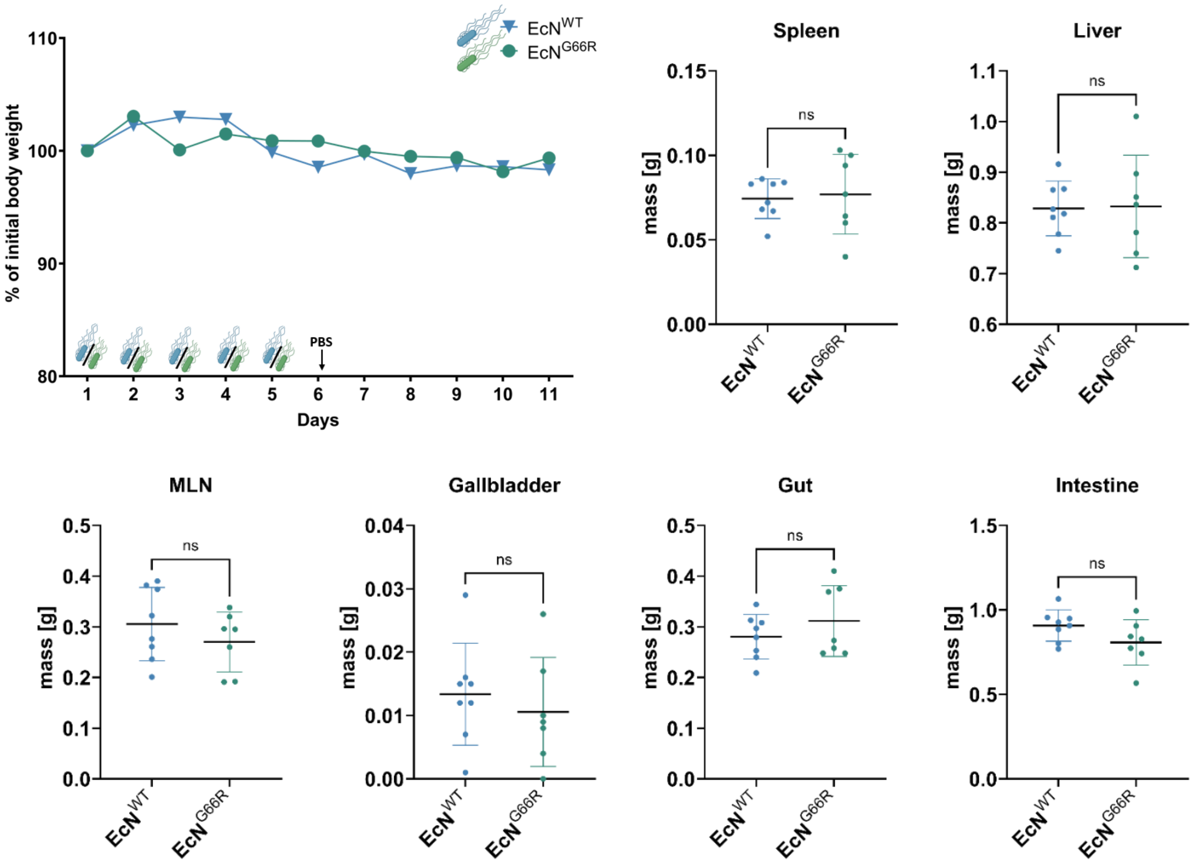

Supplementary Figure 6B: Body weight changes and organ masses following oral administration of EcN^{WT}(blue) or EcN^{G66R} (green) to BALB/c mice. (Top left) . Each data point represents an individual mouse, and horizontal lines indicate the mean ± SEM. No statistically significant differences were observed between treatment groups for any organ (ns-not significant; unpaired Student's t-test).

Supplementary Figure 6C

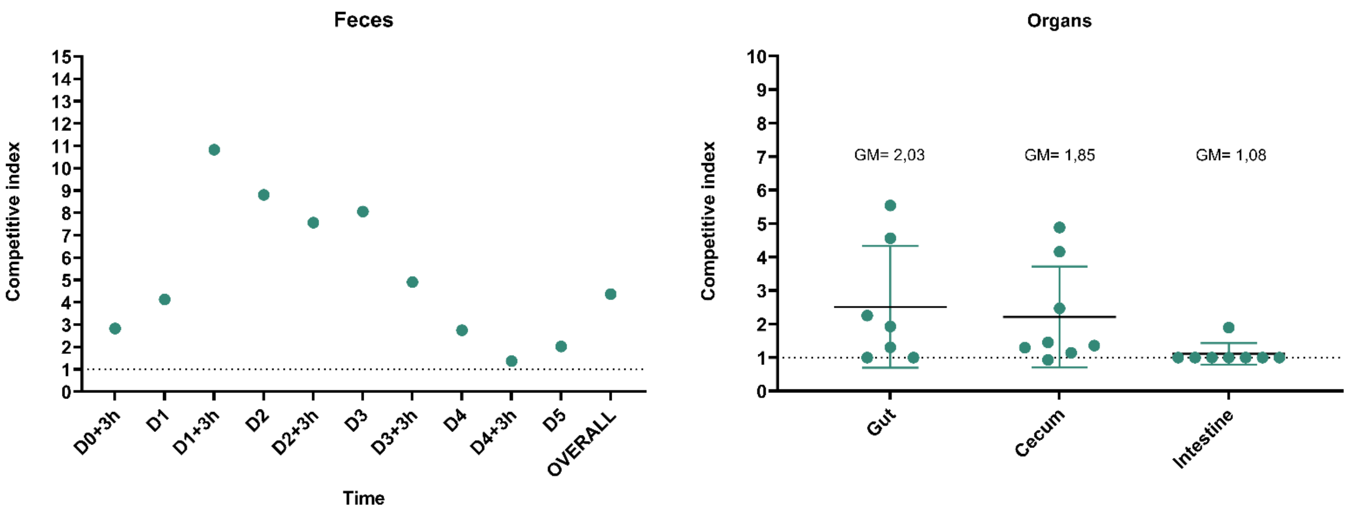

Supplementary Figure 6C. Competitive colonization index of EcN^{G66R} relative to EcN^{WT} in feces and intestinal tissues. **Feces (left panel):** geometric mean CI values at successive time points from day 0 (3 hours post-administration, D0+3h) through day 5 (D5), with an overall geometric mean. **Organs (right panel)**: CI values from gut, cecum, and intestine. Each point represents an individual mouse, and horizontal lines indicate the mean ± SD. Geometric means (GM) are annotated for each tissue. The dotted line at CI = 1 indicates equal recovery of both strains. Values consistently above 1 indicate preferential recovery of EcN^{G66R} throughout the experiment.

Supplementary Figure 6D

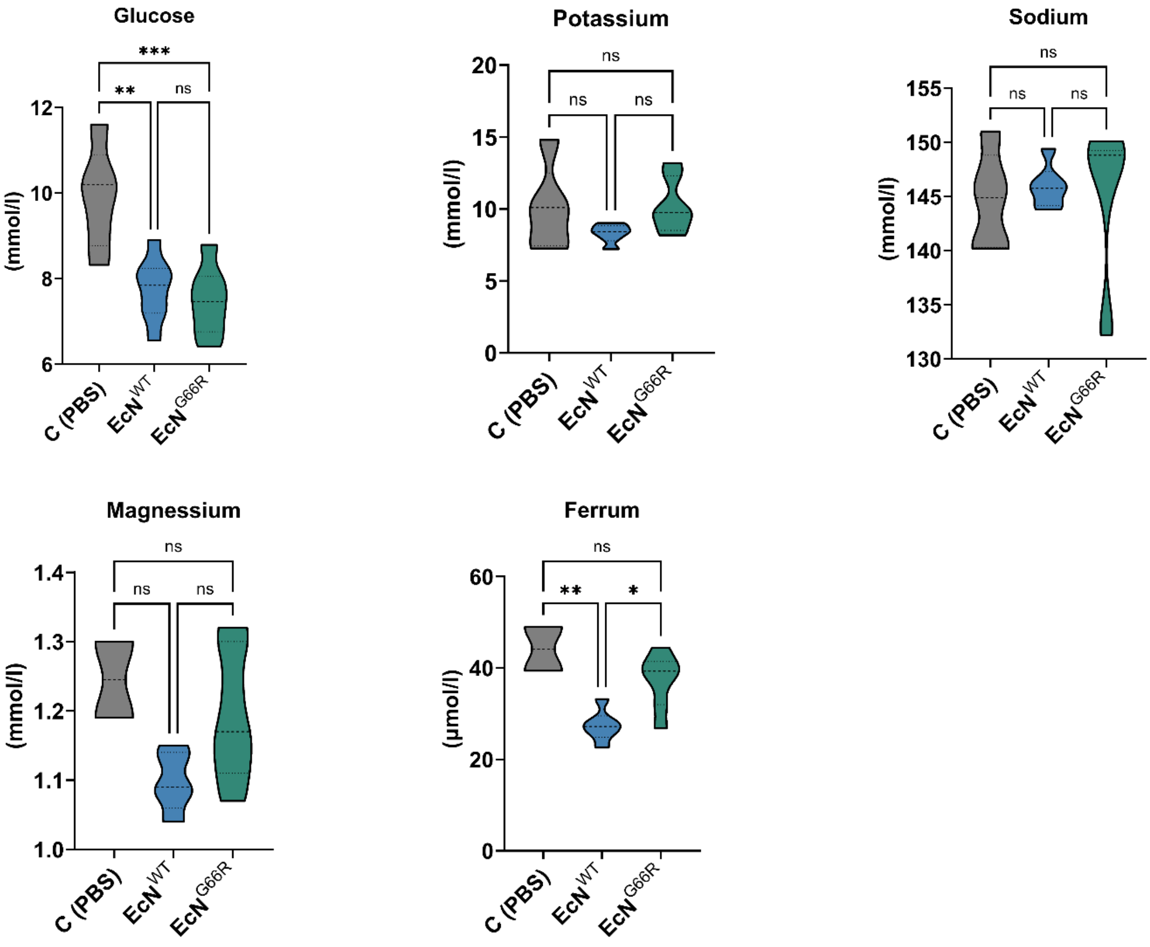

Supplementary Figure 6D. Blood biochemical parameters in BALB/c mice after oral administration of probiotic strains depicted as violin plots illustrated selected biochemical markers in mice treated with PBS (control, grey), EcN^{WT} (blue), or EcN^{G66R} mutant (green). ***p<0.001 **p<0.01 *p<0.05. Statistical analysis was performed using one-way ANOVA with Tukey's post-hoc test (glucose, potassium, magnesium, ferrum) or Kruskal-Wallis test with Dunn's post-hoc test (sodium).

Supplementary Figure 6E

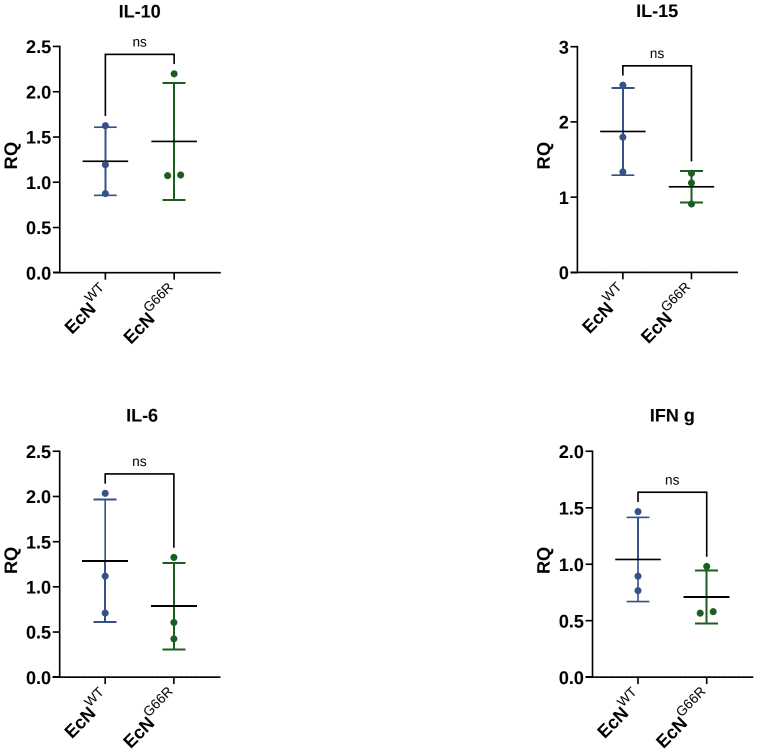

Supplementary Figure 6E. Cytokine gene expression in spleens of BALB/c mice after oral administration of EcN^{WT} (blue) or EcN^{G66R} mutan (green). Statistical analysis was conducted using unpaired Welch's test (IL-15, IL-6, IFN-γ) or unpaired t-test or Mann-Whitney test (IL-10).

Supplementary Figure 6F

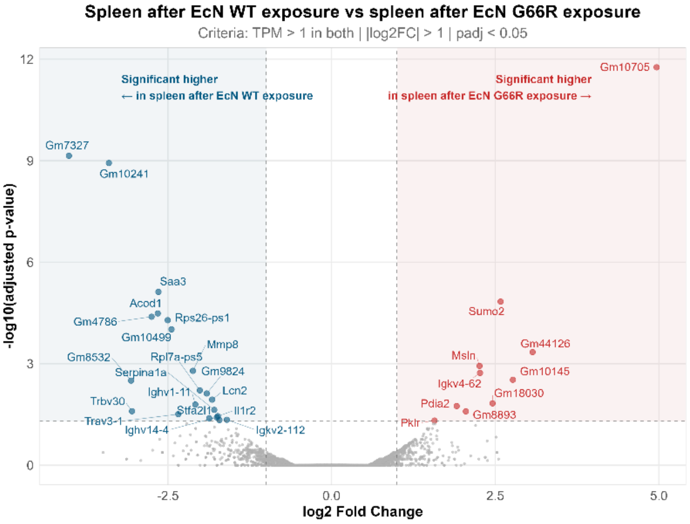

Supplementary Figure 6F. Volcano plot showing DEGs between spleens from mice pretreated with EcN^{WT} versus EcN^{G66R}. Genes with TPM > 1 in both groups, |$\log_2$(fold change)| > 1 and adjusted P-value < 0.05 are plotted. Genes significantly up-regulated (red) (right side) and genes significantly down-regulated (blue) (left side). Not significant genes – grey. Gene labels are displayed for the most significantly regulated genes (top 20 by p-value). Vertical dashed lines indicate the ±1 $\log_2$FC threshold, and the horizontal dashed line indicates the -$\log_{10}$(padj) threshold.

Supplementary Figure 6G

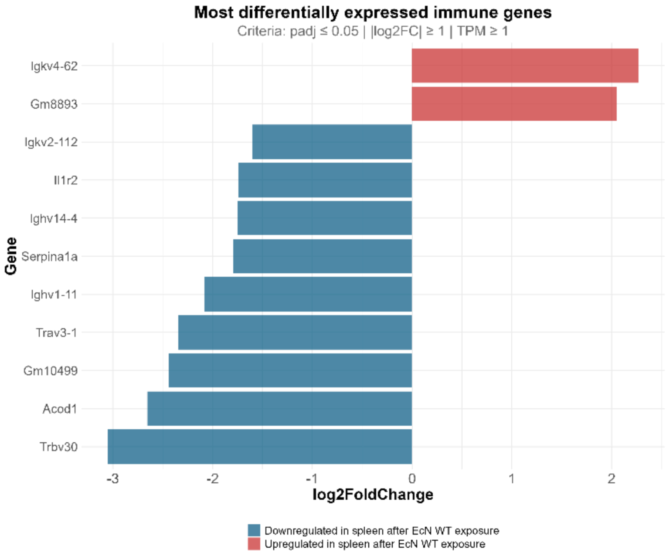

Supplementary Figure 6G. Top differentially expressed immunological genes identified by RNA-seq analysis in spleens from mice pretreated with EcN^{WT} versus EcN^{G66R}. The bar plot displays immune-related genes (2 upregulated (red) and 9 downregulated (blue)), with the highest absolute $\log_2$ fold change.

Supplementary Figure 7A

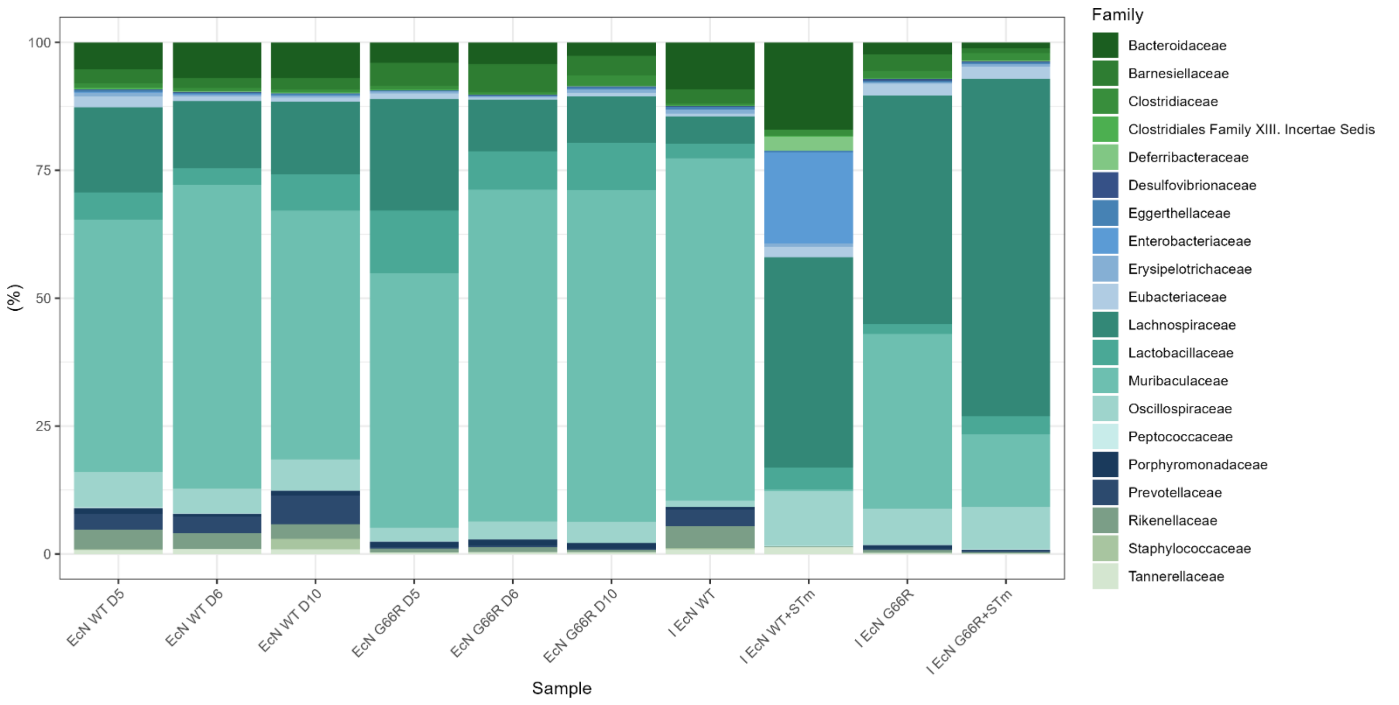

Supplementary Figure 7A. Gut microbiota composition at the family level. Relative abundance of bacterial families determined by 16S rRNA gene sequencing of fecal and intestinal tissue samples. Fecal samples were collected at day 5 (D5) of probiotic administration, day 6 (D6, one day after stopping probiotic treatment), and day 10 (D10, five days after stopping probiotic treatment) from mice treated with EcN^{WT} or EcN^{G66R}. Intestinal tissue samples (I) were collected during necropsy from uninfected probiotic-treated mice (I EcN^{WT}, I EcN^{G66R}) and from mice challenged with STm (I EcN^{WT} +STm, I EcN^{G66R} +STm), six days post-infection. Each bar represents a pooled sample from the respective experimental group.

Supplementary Figure 7B

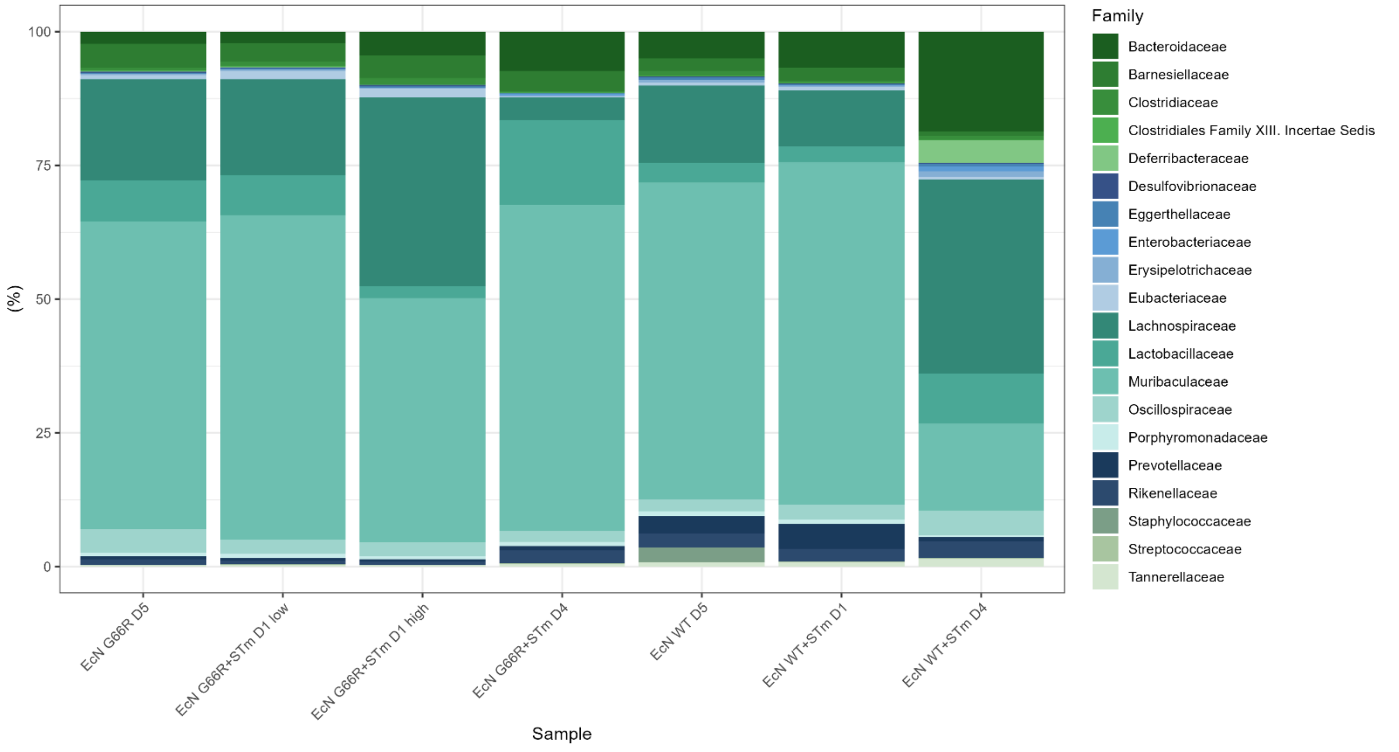

Supplementary Figure 7B. Fecal microbiota composition at the family level. Relative abundance of bacterial families determined by 16S rRNA gene sequencing of fecal samples collected from EcN^{WT}- and EcN^{G66R}-pretreated mice followed by STm challenge at day 5 of probiotic administration (D5, before pathogen challenge), day 1 post-infection (D1, shown as two separate biological replicates for EcN^{G66R} +STm: D1_low and D1_high), and day 4 post-infection (D4). Each bar represents a pooled sample from the respective experimental group.

Supplementary Table 1

Mutation	Sequence (5´−3´)	Primer name	Description
**G66R**	GAGGCTCGGCTTATGGCcgcGTGTTATCTAATTTTTCC	G66R_F	Primer introducing a point mutation
	GGAAAAATTAGATAACACgcgGCCATAAGCCGAGCCTC	G66R_R	Primer introducing a point mutation
	CGgaattcGCATATCAATGCCACGGCTACC	G66R_F_EcoRI	Primer introducing site for restriction enzymes (EcoRI)
	CGggatccGCATGGTAGCGTAAAAGAGCACC	G66R_R_BamHI	Primer introducing site for restriction enzymes (BamHI)
	CCGCGCTTTCGTGGACGACCCTGACGCCGG	G66R_F_2	Flanking primer
	GCGACGGCTGCGGGTTCCAACACCCTCACC	G66R_R_2	Flanking primer
**T158P**	GATGTGGTGGTGCCTcctGGCGGCTGCGATG	T158P_F	Primer introducing a point mutation
	CATCGCAGCCGCCaggAGGCACCACCACATC	T158P_R	Primer introducing a point mutation
	CGgaattcGCGCTTGAGTTGACTAATTGTCCG	T158P_F_EcoRI	Primer introducing site for restriction enzymes (EcoRI)
	CGggatccCACTGTGCTAATTCGTCTGGCG	T158P _R_BamHI	Primer introducing site for restriction enzymes (BamHI)
	GGAGATGCTCATGAAATGGCGCAAACG	T158P_F_2	Flanking primer
	CCTGCCTGGGAAGTGGTTAACTTTATCGG	T158P_R_2	Flanking primer
**Y186R**	GCCAATTCCTCTTACCGTTcgtTGTGCGAAAAGCCAAAACC	Y186R_F	Primer introducing a point mutation
	GGTTTTGGCTTTTCGCACAacgAACGGTAAGAGGAATTGGC	Y186R_R	Primer introducing a point mutation
	CGgaattcGCGCTTGAGTTGACTAATTGTCCG	T158P_F_EcoRI	Primer introducing site for restriction enzymes (EcoRI)
	CGggatccCACTGTGCTAATTCGTCTGGCG	T158P _R_BamHI	Primer introducing site for restriction enzymes (BamHI)
	GGAGATGCTCATGAAATGGCGCAAACG	T158P_F_2	Flanking primer
	CCTGCCTGGGAAGTGGTTAACTTTATCGG	T158P_R_2	Flanking primer

Supplementary Table 1. Primers used for generation of *fimH* point mutants in *E. coli* Nissle 1917

Supplementary Table 2

Gene	Sequence (5´−3´)	Source
IL-1B_F	CTCTCCAGCCCAAGCTTCCTTGTGC	[1]
IL-1B_R	GCTCTCATCAGGACAGCCCAGGT	[1]
IL-6_F	AAGTGCATCATCGTTGTTCATACA	This study
IL-6_R	GAGGATACCACTCCCAACAGACC	This study
IL-10_F	ATTTGAATTCCCTGGGTGAGAAG	This study
IL-10_R	CACAGGGGAGAAATCGATGACA	This study
IL-12a_F	GGAAGCACGGCAGCAGAATA	[1]
IL-12a_R	AACTTGAGGGGAGAATGAGGAATGG	[1]
IL-15_F	TCCTGCAAGTCTCTCCCAATTC	This study
IL-15_R	TTCTTTCCTGACCTCTCTGAGC	This study
IL-19_F	CTGGAAGATGACAGACAGTCTAGG	This study
IL-19_R	GGCGCATGTCCACAGAAATC	This study
INFg_F	TCAAGTGGCATAGATGTGGAAGAA	[2]
INFg_R	TGGCTCTGCAGGATTTTCATG	[2]
Actb_F	GGCTGTATTCCCCTCCATCG	[1]
Actb_R	CCAGTTGGTAACAATGCCATGT	[1]

Supplementary Table 2. Primers used for qPCR

1. Perez-Lopez, A. et al. CRTAM Shapes the Gut Microbiota and Enhances the Severity of Infection. The Journal of Immunology 203, 532–543 (2019).

2. Walker, G. T., Gerner, R. R., Nuccio, S. P. & Raffatellu, M. Murine Models of Salmonella Infection. Curr Protoc 3, e824 (2023).

Supplementary Table 3

Target	Conjugate	Catalog numer	Company
CD3	APC/Cyanine7	100222	Biolegend
CD4	PerCP/Cyanine5.5	116012	Biolegend
CD11c	PE	117308	Biolegend
CD103	APC	110906	Biolegend
CD8a	PE/Cyanine7	100722	Biolegend
CD206	PE/Cyanine7	141720	Biolegend
CD86	PerCP/Cyanine5.5	159212	Biolegend
F4/80	APC	123116	Biolegend
KI CD206	PE/Cyanine7	400522	Biolegend
KI CD86	PerCP/Cyanine5.5	400532	Biolegend

Supplementary Table 3. List of antibodies used for flow cytometry

Supplementary Table 4

	Unique upregulated genes for EcN WT	Common downregulated genes
1	Gm14794	Rps26-ps1
2	Gm31651	Aqp5
3	Prr7	Rasl11b
4	Gm3086	H4c12
5	Layn	Wnt16
6	Gm24091	Pcdh19
7	Gm49506	Pde4c
8	1110003F10Rik	H1f2
9	Snord17	Spata2l
10	Ptpn22	Arhgap40
11	Stambp-ps1	Gm8113
12	Gm20559	ENSMUSG00000120036
13	Gm14137	Scx
14	Oasl2	Spns3
15		Fgf18
16		Mturn

Supplementary Table 4. Unique upregulated genes in EcN^{WT}-treated MIEC cells and common downregulated genes across both treatments (EcN^{WT}-treated MIEC and EcN^{G66R}-treated MIEC).

Supplementary Table 5

The table presents 16S rRNA gene sequencing for three bacterial taxa: *Enterobacteriaceae* (family level), *Salmonella* (genus level), and *Escherichia* (genus level). Results are expressed as both the number of sequence reads and the relative abundance (percentage of total reads in the sample).

	Enterobacteriaceae		***Salmonella***		***Escherichia***	
	reads	%	reads	%	reads	%
**EcN^{WT} D5**	27	0.04565	2	0.003473	6	0.01042
**EcN^{WT} +STm_D1**	46	0.07792	6	0.01039	6	0.01039
**EcN^{WT} +STm_D4**	536	0.913	335	0.5823	34	0.0591
**EcN^{G66R} D5**	21	0.03562	NA	NA	3	0.005219
**EcN^{G66R} +STm_D1_low**	25	0.04231	NA	NA	5	0.008676
**EcN^{G66R} STm_D1_high**	21	0.03567	NA	NA	4	0.00702
**EcN^{G66R} STm_D4**	125	0.2107	88	0.1503	2	0.003416

Supplementary Table 5A. **Fecal samples of BALB/c mice collected at different time points during probiotic administration and *Salmonella* Typhimurium infection**. Samples were collected at the following time points: D5—after 5 days of probiotic administration (before pathogen challenge); D1—one day after STm oral gavage; D4—four days after STm oral gavage. The table includes data from mice pretreated with EcN^{WT} or EcN^{G66R} probiotic strains. For EcN^{G66R} +STm D1, two biological replicates are shown separately (D1_low and D1_high). NA indicates samples where no reads for the specified taxon were detected.

	Enterobacteriaceae		***Salmonella***		***Escherichia***	
	reads	%	reads	%	reads	%
**EcN^{WT}**	15	0.0261	NA	NA	NA	NA
**EcN^{WT} + STm**	9300	17.55	3997	8.391	476	0.9992
**EcN^{G66R}**	17	0.0296	NA	NA	3	0.005477
**EcN^{G66R} + STm**	15	0.02618	3	0.005554	NA	NA

Supplementary Table 5B. Intestinal tissue samples collected during necropsy of BALB/c mice. Tissue samples were collected 6 days post-infection (or at the equivalent time point for uninfected controls). The table includes data from mice pretreated with EcN^{WT} or EcN^{G66R} probiotic strains, with or without subsequent STm challenge. NA indicates samples where no reads for the specified taxon were detected.

	Enterobacteriaceae		***Salmonella***		***Escherichia***	
	reads	%	reads	%	reads	%
**EcN^{WT} D5**	19	0.03283	NA	NA	NA	NA
**EcN^{WT} D6**	19	0.03276	NA	NA	1	0.00176
**EcN^{WT} D10**	40	0.06905	NA	NA	1	0.001768
**EcN^{G66R} D5**	13	0.02221	NA	NA	NA	NA
**EcN^{G66R} D6**	11	0.01878	NA	NA	NA	NA
**EcN^{G66R} D10**	15	0.02561	NA	NA	1	0.001748

Supplementary Table 5C. Fecal samples of BALB/c mice receiving probiotic treatment without subsequent pathogen challenge. Samples were collected at the following time points: D5—after 5 days of probiotic administration; D6—one day after stopping of probiotic administration (6 days from first administration); D10—five days after stopping of probiotic administration (10 days from first administration). The table includes data from mice treated with EcN^{WT} or EcN^{G66R} probiotic strains. NA indicates samples where no reads for the specified taxon were detected.

Supplementary Table 6

	Genes with significantly higher expression in EcN^{WT}-treated cells	Genes with significantly higher expression in EcN^{G66R}-treated cells
1	Gm14794	Gm17082
2	Gm6741	Apoo-ps
3	Gm29770	Snord98
4	ENSMUSG00000120250	Gm50023
5	1700039E22Rik	Gapdh
6	Tmed6	Gm6627
7	Gm12115	ENSMUSG00000120514
8	Gm2962	Gm6397
9	Rgs2	4833412K13Rik
10	Etfbkmt	Gm8129
11	ENSMUSG00000120717	Gm6254
12	Rab3a	Gm43272
13	Gsta1	Gm5854
14	Mir181a-1hg	Gm10123
15	Hmgn2-ps	
16	Gm28438	
17	Tert	

Supplementary Table 6. Differentially expressed genes in MIEC cells following stimulation with EcN^{WT} or EcN^{G66R}.

Supplementary Table 7

	Genes with significantly downregulated expression in EcN^{G66R}	Genes with significantly higher expression in EcN^{G66R}
1	Gm10705	Gm7327
2	Gm44126	Gm10241
3	Gm10145	Gm8532
4	Sumo2	Trbv30
5	Gm18030	Gm4786
6	Igkv4-62	Acod1
7	Msln	Saa3
8	Gm8893	Rps26-ps1
9	Pdia2	Gm10499
10	Pklr	Trav3-1
11		Mmp8
12		Ighv1-11
13		Rpl7a-ps5
14		Gm9824
15		Stfa2l1
16		Lcn2
17		Serpina1a
18		Ighv14-4
19		Il1r2
20		A130071D04Rik
21		Igkv2-112

Supplementary Table 7. Differentially expressed genes in spleens of BALB/c mice following colonization with EcN^{G66R} compared to EcN^{WT}.
